# Immune evolution from preneoplasia to invasive lung adenocarcinomas and underlying molecular features

**DOI:** 10.1038/s41467-021-22890-x

**Published:** 2021-05-11

**Authors:** Hitoshi Dejima, Xin Hu, Runzhe Chen, Jiexin Zhang, Junya Fujimoto, Edwin R. Parra, Cara Haymaker, Shawna M. Hubert, Dzifa Duose, Luisa M. Solis, Dan Su, Junya Fukuoka, Kazuhiro Tabata, Hoa H. N. Pham, Nicholas Mcgranahan, Baili Zhang, Jie Ye, Lisha Ying, Latasha Little, Curtis Gumbs, Chi-Wan Chow, Marcos Roberto Estecio, Myrna C. B. Godoy, Mara B. Antonoff, Boris Sepesi, Harvey I. Pass, Carmen Behrens, Jianhua Zhang, Ara A. Vaporciyan, John V. Heymach, Paul Scheet, J. Jack Lee, Jia Wu, P. Andrew Futreal, Alexandre Reuben, Humam Kadara, Ignacio I. Wistuba, Jianjun Zhang

**Affiliations:** 1 Department of Translational Molecular Pathology, the University of Texas MD Anderson Cancer Center, Houston, TX USA; 2 Department of Genomic Medicine, the University of Texas MD Anderson Cancer Center, Houston, TX USA; 3 Department of Thoracic/Head and Neck Medical Oncology, the University of Texas MD Anderson Cancer Center, Houston, TX USA; 4 Department of Bioinformatics & Computational Biology, the University of Texas MD Anderson Cancer Center, Houston, TX USA; 5 Institute of Cancer and Basic Medicine (IBMC), Chinese Academy of Sciences, Hangzhou, China; 6 Department of Pathology, Cancer Hospital of the University of Chinese Academy of Sciences (Zhejiang Cancer Hospital), Hangzhou, China; 7 Department of Pathology, Kagoshima University Graduate School of Medical and Dental Sciences, Kagoshima, Japan; 8 Department of Pathology, Nagasaki University Graduate School of Biomedical Sciences, Nagasaki, Japan; 9 Cancer Research United Kingdom-University College London Lung Cancer Centre of Excellence, London, UK; 10 Zhejiang Cancer Research Institute, Cancer Hospital of the University of Chinese Academy of Sciences (Zhejiang Cancer Hospital), Hangzhou, China; 11 Department of Epigenetics and Molecular Carcinogenesis, the University of Texas MD Anderson Cancer Center, Houston, TX USA; 12 Center of Cancer Epigenetics, the University of Texas MD Anderson Cancer Center, Houston, TX USA; 13 Department of Thoracic Imaging, the University of Texas MD Anderson Cancer Center, Houston, TX USA; 14 Department of Thoracic and Cardiovascular Surgery, the University of Texas MD Anderson Cancer Center, Houston, TX USA; 15 Department of Cardiothoracic Surgery, New York University Langone Medical Center, New York, NY USA; 16 Department of Epidemiology, the University of Texas MD Anderson Cancer Center, Houston, TX USA; 17 Department of Biostatistics, the University of Texas MD Anderson Cancer Center, Houston, TX USA; 18 Department of Imaging Physics, The University of Texas MD Anderson Cancer Center, Houston, TX USA

**Keywords:** Non-small-cell lung cancer, Evolutionary genetics, Immunoediting, Non-small-cell lung cancer, Immunoediting

## Abstract

The mechanism by which anti-cancer immunity shapes early carcinogenesis of lung adenocarcinoma (ADC) is unknown. In this study, we characterize the immune contexture of invasive lung ADC and its precursors by transcriptomic immune profiling, T cell receptor (TCR) sequencing and multiplex immunofluorescence (mIF). Our results demonstrate that anti-tumor immunity evolved as a continuum from lung preneoplasia, to preinvasive ADC, minimally-invasive ADC and frankly invasive lung ADC with a gradually less effective and more intensively regulated immune response including down-regulation of immune-activation pathways, up-regulation of immunosuppressive pathways, lower infiltration of cytotoxic T cells (CTLs) and anti-tumor helper T cells (Th), higher infiltration of regulatory T cells (Tregs), decreased T cell clonality, and lower frequencies of top T cell clones in later-stages. Driver mutations, chromosomal copy number aberrations (CNAs) and aberrant DNA methylation may collectively impinge host immune responses and facilitate immune evasion, promoting the outgrowth of fit subclones in preneoplasia into dominant clones in invasive ADC.

## Abstract (web summary)

The evolution of immune landscape in lung adenocarcinoma (ADC) is largely unknown. Here the authors use a cohort of resected invasive lung ADC and its precursors and show a gradual increase of immunosuppression and decrease of anti-tumor response associated with specific genomic and epigenetic features.

## Introduction

Despite promising advances in clinical management, lung cancer remains the leading cause of cancer death worldwide, largely due to diagnosis at advanced stages when cures are generally unachievable. Computed tomography (CT) scan-guided lung cancer screening has demonstrated a reduction of lung cancer mortality by 26–61%^1^, underscoring the need for early detection and intervention as a crucial strategy to reduce lung cancer incidence and mortality. However, the outcomes of many randomized clinical trials on primary lung cancer prevention have been disappointing^1^, primarily due to our rudimentary knowledge of early phases in lung cancer development. In depth understanding of the molecular mechanisms of early lung carcinogenesis may accelerate the development of precise diagnostic as well as effective preventive and therapeutic strategies.

Lung adenocarcinoma (ADC) is the most common histological subtype of lung cancer. The classification of early-stage lung ADC and its precursors has been revised by several professional societies to include a spectrum from atypical adenomatous hyperplasia (AAH), the only recognized preneoplasia to lung ADC, to preinvasive adenocarcinoma in situ (AIS), to micro-invasive lesions termed minimally invasive adenocarcinoma (MIA), and eventually frankly invasive ADC^2–5^. Early-stage lung ADCs and their precursors usually present as lung nodules with distinct radiologic features called ground glass opacity (GGO). These lung nodules are often referred to as indeterminate pulmonary nodules (IPNs) without histologic diagnosis, as the diagnostic yield from biopsy of GGO-predominant nodules is low and surgical resection is not the standard of care. This has subsequently led to scarcity of adequate materials to investigate the molecular landscape of lung ADC precursors.

**Fig. 1:**
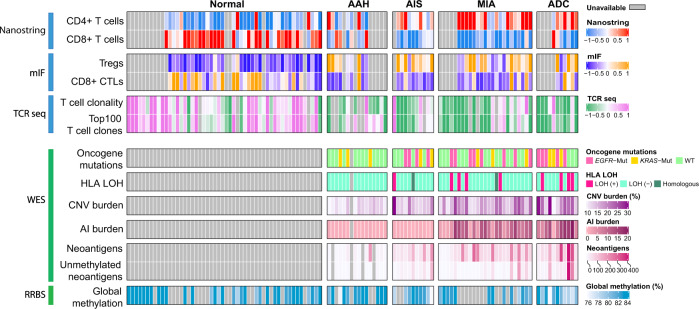
The immune landscape IPNs of different stages and associated genomic and epigenomic features. Infiltration CD4+ T cells, CD8+ T cells inferred from immune gene expression using TIMER; regulatory T cells (Tregs, CD3+CD8–FOXP3+) and CD8+cytotoxic T lymphocytes (CTLs,CD3+CD8+granzyme B+) measured by multiplex immunofluorescence (mIF); T cell clonality and frequency of the top 100T cell clones by TCR sequencing are shown in upper panel. Genomic alterations from whole exome sequencing (WES) including *EGFR*/*KRAS* mutations, HLA LOH, copy number variation (CNV) burden, allelic imbalance (AI) burden, total number of mutations associated with predicted neoantigens, and total number of mutations associated with predicted neoantigens without promoter methylation; global methylation status using long interspersed transposable elements-1 (LINE-1) as a surrogate marker accessed by reduced representation bisulfite sequencing (RRBS) are shown in bottom panel. Data from 53 patients was used including AAH (typical adenomatous hyperplasia), AIS (adenocarcinoma in situ), MIA (minimally invasive adenocarcinoma), and ADC (invasive adenocarcinoma). (Source data is provided as a source data file).

Carcinogenesis results from progressive accumulation of molecular abnormalities (molecular evolution) and escape from host immune surveillance (immunoediting). Our pilot studies of gene expression and genomic profiling on lung ADC precursors have demonstrated distinct transcriptomic features^6^ and progressive genomic evolution along the spectrum of AAH to AIS, MIA, and ADC^7^. However, the extent to which immunoediting sculpts early carcinogenesis of lung ADC and the underlying genomic and epigenetic alterations associated with these immune features still remain undetermined. In the current study, we perform immune gene expression profiling, T cell receptor (TCR) sequencing and multiplex immunofluorescence (mIF) staining on a cohort of resected AAH, AIS, MIA and invasive ADC lesions and paired morphologically normal lung tissues (NL) to delineate the evolution of immune contexture, particularly the T cell landscape, across different stages of early lung ADC pathogenesis. We further leverage whole exome sequencing (WES)^7^ and methylation data^8^ from the same cohort of IPNs to unravel the genomic and methylation alterations that may impinge on the immune contexture (Supplementary Data 1 and Fig. 1).

## Results

### Decreased overall immunity in invasive lung adenocarcinomas compared to their precursors

**Fig. 2:**
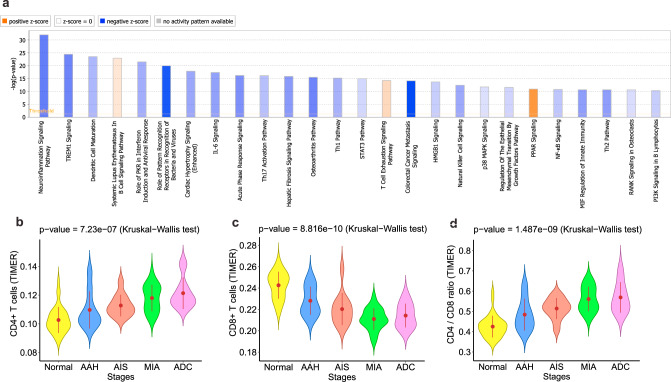
Immune landscape from preneoplasia to invasive lung adenocarcinoma. **a** Significantly enriched functional pathways based on the 291 differentially expressed genes by Ingenuity Pathway Analysis (IPA®; Ingenuity Systems) software. Pathways with –log (*p*-value) > 10 (*p*-values are obtained from Fisher’s right-tailed exact test) and an absolute *z*-score > 0.5 are shown. Pathways that were predicted to be inhibited (negative *Z* scores) in later stages are in blue and pathways that were predicted to be activated (positive *Z* scores) in later stages are in orange. The heights of the bars indicate the significance of the enrichment (−log (*p*-value)) and the scales of the orange or blue colors represent the predicted directionality. Fractions of immune cells including CD4+ T cells (**b**), CD8+ T cells (**c**), and CD4/CD8 ratio (**d**) were estimated using TIMER based on the gene expression using nCounter PanCancer Immune Profiling Panel. Error bars indicate 95% confidence intervals and solid point represent mean value in each stage. The difference of cell fraction among different stages was evaluated using two-sided Kruskal–Wallis H test. Data from 38 patients was used including NL (normal lung tissue), AAH (atypical adenomatous hyperplasia), AIS (adenocarcinoma in situ), MIA (minimally invasive adenocarcinoma), and ADC (invasive adenocarcinoma). (Source data is provided as a source data file).

To assess the dynamic changes in the immune contexture during early lung carcinogenesis, we performed immune profiling using the nCounter PanCancer Immune Profiling Panel (NanoString Technologies, Inc.) composed of 770 genes including ~700 immune-related genes covering both innate and adaptive immune response^9^ on resected lung ADCs and their precursors (*n* = 9 for AAH, *n* = 11 for AIS, *n* = 21 for MIA, and *n* = 6 for invasive ADC) (Supplementary Data 2). There was no difference in age (*p* = 0.55, Kruskal–Wallis test), sex (*p* = 0.31, Fisher’s exact test) or smoking status (*p* = 0.35, Fisher’s exact test) between patients with pulmonary nodules of different histologic stages. A total of 291 differentially expressed genes (DEGs; FDR < 0.1%) were identified. Interestingly, changes in the majority of DEGs, regardless of their directions, exhibited a progressive pattern along the spectrum from NL, to AAH, AIS, MIA, and ADC (Supplementary Data 3). Examples of progressively increased genes included immune suppressive genes *CD47* (protection of cancer cells from immune cell killing)^10^, *CD276* (inhibition of immune responses)^11^ and *CTLA4* (checkpoint molecule)^12^, while progressively decreased genes included *ENTPD1* (expressed on tumor-specific T cells)^13^, granzyme B (*GZMB*), and perforin 1 (*PRF1*) (cytotoxic molecules produced by T lymphocytes and natural killer cells)^14,15^ (Supplementary Fig. 1). Functional pathway analysis of these 291 DEGs revealed 26 significantly de-regulated pathways (–log(*p*-value) > 10 and an absolute *z*-score > 0.5) associated with neoplastic evolution from NL to invasive ADC, of which, 23 were down-regulated (Fig. 2a) in later-stage IPNs. On the other hand, all three up-regulated pathways in later-stage IPNs (systemic lupus erythematosus (SLE) in B cell signaling, T cell exhaustion signaling, and PARP signaling pathways) could potentially impair immune response^16–18^. These results indicated that immune evolution progressed as a continuum from preneoplasia to invasive lung ADC with an overall decreased immunity in later-stage diseases.

### Intensively regulated anti-tumor immune response in invasive lung adenocarcinomas versus their precursors

We next de-convoluted immune gene expression profiling data using TIMER^19^ to evaluate the changes in immune cell composition. As shown in Fig. 2b, CD4+ T lymphocyte infiltration progressively increased from NL to invasive ADC. Conversely, infiltration of CD8+ T lymphocytes progressively decreased with neoplastic evolution (Fig. 2c) leading to significantly higher CD4/CD8 ratio in later-stage lesions (Fig. 2d). To validate these findings, we applied TIMER to RNA sequencing (RNA-seq) data from a previously published independent cohort of lung ADC precursors^6^ and observed similar results (Supplementary Fig. 2).

Although CD8+ T cells are widely accepted to be anti-tumor^20^, the functions of CD4+ T cells are more complicated. CD4+ T cells are highly heterogeneous in phenotype, and classified into T helpers including T helper 1 (Th1, anti-viral/anti-tumor), T helper 2 (Th2, allergic responses), T helper 17 (Th17, auto-immune and pathogen responses), and Tregs (immune suppressive)^21–24^. Recent work has also described populations of CD4+ T cells with cytotoxic potential (ThCTLs), which could be beneficial to anti-tumor responses^25^. To characterize the phenotype of infiltrated T cells in these IPNs, we assessed T cell markers by mIF (Supplementary Table 1 and Supplementary Fig. 3) on a subset of IPNs with materials available. As anti-CD4 antibodies are known to be problematic for mIF^26^, CD3+/CD8– T cells were used to represent CD4+ T cells for mIF. As shown in Supplementary Fig. 4, the proportion of CD4+ T cells inferred by immune gene expression profiling was positively associated with total T cells (CD3+), Tregs (CD3+CD8–FoxP3+), but not with ThCTLs (CD3+CD8–GZMB+) from mIF. Conversely, CD8+ T cells inferred by immune gene expression profiling were positively associated with CD8+CTLs (CD3+CD8+GZMB+), but negatively associated with Tregs (CD3+CD8–FoxP3+) by mIF. The CD4/CD8 ratio inferred by gene expression data was also positively associated with Treg/CD8+CTL (CD3+CD8−FoxP3+/CD3+CD8+GZMB+) ratio by mIF. Despite ThCTLs (CD3+CD8–GZMB+) and Tregs (CD3+CD8–FoxP3+) only representing a minority of CD4+ T cells in these IPNs (Supplementary Fig. 5), normal lung tissues had the highest proportion of ThCTLs (Supplementary Fig. 5a) and the lowest proportion of Tregs (Supplementary Fig. 5b) indicating that immune evasion may have started as early as the preneoplastic stage. Of note, though present at low abundance, Tregs increased in later-stage IPNs indicating further negative immune regulation along with neoplastic evolution (Supplementary Fig. 5b).

In addition, we attempted to assess T helper polarization in different stages of IPNs. Due to limited cell surface markers tested in the current mIF panel, we derived T helper signatures using gene expression data. These analyses revealed that the ratios of Th2/Th1 and Th17/Th1 are significantly higher in later-stage IPNs (Supplementary Fig. 6a, b) suggesting a skewing of T cells towards a less anti-tumor phenotype, though functional studies are needed to confirm these observations. Moreover, ratios of Th2/Th1 and Th17/Th1 derived from RNA-seq data of an independent cohort of AAH and ADC (GSE102511)^6^ revealed the same trend (Supplementary Fig. 6c, d). Collectively, these results suggest that immune evolution progressed from preneoplasia to invasive lung ADC with potential loss of anti-tumor responses and gain of suppressive or ineffective subsets of immune cells.

### Progressively divergent TCR repertoire with neoplastic progression

**Fig. 3:**
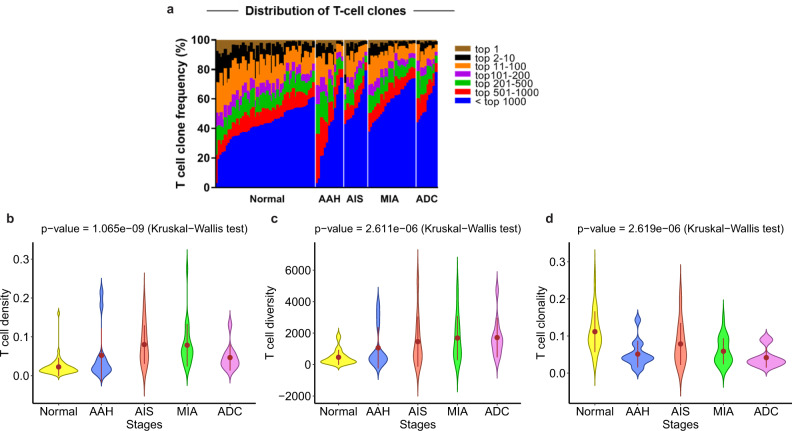
T cell repertoire from preneoplasia to invasive lung adenocarcinoma. **a** Distribution of T cell clones with frequency of top 1 (brown), top 2–10 (black), top 11–100 (orange), top 101–200 (purple), top 201–500 (green), top 501–1000 (red), and beyond 1000 (blue) in normal lung, AAH, AIS, MIA, and invasive ADC lesions. **b** T cell density, **c** T cell diversity, and **d** T cell clonality in normal lung, AAH, AIS, MIA, and invasive ADC. Error bars indicate 95% confidence intervals and solid point represent mean value in each stage. The difference of T cell matrix among different stages was evaluated using two-sided Kruskal–Wallis H test. Data from 51 patients was used including NL (normal lung tissue), AAH (atypical adenomatous hyperplasia), AIS (adenocarcinoma in situ), MIA (minimally invasive adenocarcinoma), and ADC (invasive adenocarcinoma). (Source data is provided as a source data file).

**Fig. 4:**
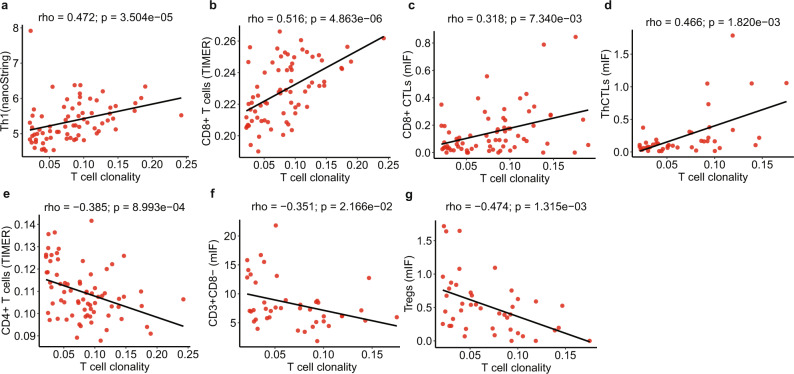
The correlation between T cell clonality and T cell subtypes. **a** Th1 (average value of marker genes IFNG, IL12A, IL12B) measured by gene expression from nCounter PanCancer Immune Profiling Panel. Fractions of CD8+ T cells (**b**) and CD4+ T cells (**e**) inferred from gene expression profiling using TIMER. **c** CD8+CTLs (CD3+CD8+granzyme B+), **d** ThCTLs (CD3+CD8–Granzyme B+), **f** CD3+CD8– T cells, and **g** Tregs (CD3+CD8–FoxP3+) measured by mIF. The correlation coefficient (rho) was assessed by two-tailed Spearman’s rank correlation test. Data from 35 patients was used including NL (normal lung tissue), AAH (atypical adenomatous hyperplasia), AIS (adenocarcinoma in situ), MIA (minimally invasive adenocarcinoma), and ADC (invasive adenocarcinoma). (Source data is provided as a source data file).

Given the central role of T cells in anti-tumor immune surveillance, we next sought to investigate the T cell repertoire^27^ by T cell receptor (TCR) sequencing and compared the IPNs of different stages for complementary TCR metrics, including top frequent T cell clones, T cell clonality, T cell density, and T cell diversity. As shown in Fig. 3a and Supplementary Fig. 7, the top T cell clones accounted for the highest proportion of T cell clones in NLs, and gradually decreased in AAH, AIS, MIA, and invasive ADC (*p* < 0.0001) implying reduced T cell expansion during early carcinogenesis of lung ADC. Moreover, NL showed the highest T cell clonality, while later-stage IPNs appeared to have higher T cell density and diversity, but lower T cell clonality (Fig. 3b–d). Of note, both T cell density and T cell diversity were positively correlated with CD4+ T cells inferred by immune gene expression profiling, as well as CD3+CD8– T cells and Tregs (CD3 + CD8–FOXP3+) by mIF (Supplementary Fig. 8). Alternatively, T cell clonality was positively associated with Th1 signature, CD8+ T cells inferred by immune gene expression, CD8+CTLs (CD3+CD8+GZMB+) and ThCTLs (CD3+CD8–GZMB+) by mIF, but negatively associated with CD4+ T cells inferred by immune gene expression, CD3+CD8− T cells and Tregs (CD3+CD8–FoxP3+) by mIF (Fig. 4).

In line with low T cell clonality generally indicating a lack of T cell expansion and a suppressed T cell repertoire^28^, T cell clonality showed significantly positive correlation with the frequency of the most dominant T cell clones in these IPNs (Supplementary Fig. 9a–d), suggesting higher T cell clonality was associated with increased clonal expansion. Conversely, T cell clonality was not correlated with T cell density (Supplementary Fig. 9e) suggesting lower T cell clonality in later-stage IPNs likely reflected reduced T cell expansion, rather than T cell exclusion. Taken together, these results suggest that later-stage IPNs had higher and more diverse T cell infiltration accompanied with reduced clonal expansion. Having also observed higher Tregs, lower CTLs and Th1 in later-stage IPNs; one plausible explanation is that although the host immune system may have recruited a greater number of T cells to IPNs with disease progression, these T cells were not able to effectively respond and expand due to increased immune suppression (e.g., increased Tregs).

### Chromosomal instability and HLA loss contributing to impaired T cell responses

**Fig. 5:**
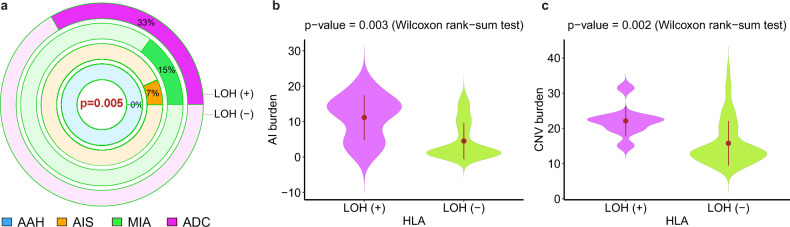
Loss of heterozygosity in HLA (HLA LOH) in IPNs of different histologic stages and its association with chromosomal alterations. **a** The proportion of AAH, AIS, MIA, and ADC lesions with evidence of HLA-LOH. Two-sided *χ*^2^ test was used to assess the difference among different histologic stages. Comparison of AI burden (number of AI events) (**b**) and CNV burden (normalized as the percent of genes with CNV) (**c**) in lesions with (purple) and without (green) HLA-LOH. Two-sided Wilcoxon rank-sum test was used to assess the differences. Error bars indicate 95% confidence intervals and solid point represent mean value in each stage. Data from 35 patients was used including NL (normal lung tissue), AAH (atypical adenomatous hyperplasia), AIS (adenocarcinoma in situ), MIA (minimally invasive adenocarcinoma), ADC (invasive adenocarcinoma). (Source data is provided as a source data file).

We next sought to explore the molecular features associated with the immune contexture observed in these lesions. Chromosomal copy number changes have been reported to impact the immune microenvironment across various cancers^29,30^ and we have observed higher copy number variation (CNV) burden and allelic imbalance (AI) burden in later-stage IPNs ADC^7^. Therefore, we sought to explore whether copy number changes were associated with immune landscape of these lung ADC precursors. As shown in Supplementary Fig. 10a–c, AI burden was negatively correlated with CD8+ T cell infiltration, while positively correlated with CD4+ T cell infiltration and CD4/CD8 T cell ratio inferred by gene expression profiling. A similar trend was also observed with CNV burden, although these differences did not reach statistical significance (Supplementary Fig. 10d–f). In light of recent studies suggesting immune evasion could be facilitated by invalid presentation of neoantigens due to loss of HLA^31^, we applied LOHHLA (loss of heterozygosity in HLA) algorithm^31^ to WES data of these lesions^7^ and observed increased incidence of LOH at the HLA loci in later-stage IPNs with 7, 15, and 33% in AIS, MIA, and ADC respectively, but none in AAH (Fig. 5a, *p* = 0.005, *χ*^2^ test). Interestingly, the IPNs with HLA LOH exhibited significantly higher AI burden and CNV burden compared to those without HLA LOH (Fig. 5b, c). One plausible explanation is that the development of AI, CNV, or HLA LOH resulted from chromosomal instability (CIN) and cells with higher levels of CIN may lead to higher CNV/AI burdens as well as increased likelihood of HLA LOH, which could subsequently enable these cells escaping from anti-tumor immune surveillance and developing into dominant clones in the later-stage neoplastic lesions.

### Aberrant DNA methylation may impact genomic alterations and anti-tumor response during early lung cancer pathogenesis

**Fig. 6:**
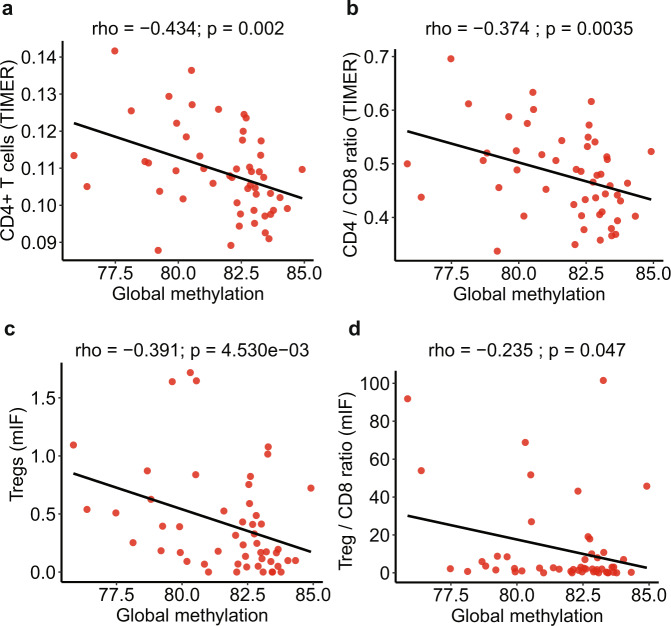
The potential impact of global methylation and immune infiltration. The correlation between global methylation levels (using LINE-1 as a surrogate marker) with CD4+ T cells (**a**), CD4/CD8 ratio (**b**) inferred from immune gene expression by TIMER as well as Treg (CD3+CD8–FOXP3+) (**c**) and Treg(CD3+CD8−FOXP3+)/CD8(CD3+CD8+) ratio (**d**) measured by mIF. The correlation coefficient (rho) was assessed by two-tailed Spearman’s rank correlation test. Data from 24 patients was used including NL (normal lung tissue), AAH (atypical adenomatous hyperplasia), AIS (adenocarcinoma in situ), MIA (minimally invasive adenocarcinoma), and ADC (invasive adenocarcinoma). (Source data is provided as a source data file).

Somatic mutations play central roles in activating anti-tumor immune responses through generating neoantigens recognized by T cells^32,33^. As shown in Supplementary Fig. 11, a progressive increase in total mutation burden was observed from AAH to AIS, MIA, and ADC. However, analysis of methylation data from reduced representation bisulfite sequencing (RRBS) of the same cohort of IPNs^8^ revealed that a significantly larger proportion of genes exhibited promoter hypermethylation (>30% CpG methylated) in later-stage lesions, thus potentially dampening the expression of neoantigens in later-stage lesions. These data suggest that promoter hypermethylation could potentially contribute to neoantigen depletion and immune escape. Furthermore, we assessed the impact of global methylation status, using long interspersed transposable elements-1 (LINE-1) as a surrogate marker^34–36^ on the immune microenvironment in these lesions. Global hypomethylation significantly increased in IPNs of later histologic stages (Supplementary Fig. 12a) and negatively correlated with AI burden (Supplementary Fig. 12b), CNV burden (Supplementary Fig. 12c), and TMB (Supplementary Fig. 12d) indicating global hypomethylation was associated with an elevated level of CIN. Interestingly, global methylation level was negatively associated with CD4+ T cell infiltration and CD4/CD8 ratio inferred by gene expression profiling, as well as Tregs infiltration and Treg(CD3+CD8–FOXP3+)/CD8(CD3+CD8+) ratio from mIF (Fig. 6), suggesting decreased global methylation is associated with suppressive immune contexture.

### Driver mutations may affect host immune response in pre/early ADC

Cancer gene mutations are known to associate with distinct molecular landscape and tumor immune microenvironment in lung cancers. We next explored whether mutations of *EGFR* and *KRAS*, the two most frequently mutated driver genes in this cohort of IPNs^7^, were associated with the observed immune features. Compared to *EGFR-*mutant lesions or double wild-type lesions, *KRAS*-mutant IPNs exhibited the highest CD8+ T cell infiltration and lowest CD4/CD8 T cell ratio inferred by immune gene expression; lowest Treg/CD8+ T cell ratio by mIF as well as highest T cell clonality derived by TCR sequencing (Supplementary Fig. 13a–d), implying the interplay between oncogene mutations and immune surveillance during early pathogenesis of lung adenocarcinoma. Of note, the TMB and global methylation status were similar among these molecular subtypes (Supplementary Fig. 13e, f), consistent with our previous studies in invasive lung cancers^37^ and suggesting potential impact of these driver mutations on immune microenvironment independent of global genomic and methylation status.

## Discussion

The increasing implementation of CT-guided screening and advent of high-resolution diagnostic CT scans has resulted in a drastic increase in the detection of IPNs^38,39^, many of which are lung ADC precursors. The management of IPNs is now guided by a clearer clinical understanding of malignant progression. To reduce overtreatment, many IPNs are now followed by imaging surveillance without surgical intervention, unless demonstrating aggressive features such as rapid growth. Early-stage lung cancers can now be treated with minimally invasive surgical procedures that are well-tolerated with high cure rate and low cost due to brief hospital stays. On the other hand, ~20% of patients can present with multifocal diseases^40^ that may represent metachronous primary lung cancers or lung cancer precursors, which complicate the surgery. For these patients, surgical resection of a primary lung cancer could be complemented by adjuvant or chemoprevention approaches. Current treatment strategies would greatly benefit from a better understanding of the molecular mechanisms underlying early lung carcinogenesis.

Our current knowledge of early lung adenomatous carcinogenesis is rudimentary. Cancer evolution is shaped by the interaction between cancer cells and host immune surveillance, a process termed immunoediting, consisting of elimination, equilibrium, and escape phases^41^. It is well documented that the majority of human cancers are infiltrated with various immune cells, but often in an immunosuppressive microenvironment as remnant evidence of immunoediting^41^. T cell immunity is significantly compromised even in stage I lung cancers^37,42^ indicating that these cancers have already begun evading immune surveillance. However, when and how the elimination and equilibrium phases occur over the lung cancer evolution continuum is unknown. Investigating the molecular and immune landscape of lung cancer precursors is warranted to elucidate the timing of immune activation/evasion and its underlying molecular mechanisms during early lung carcinogenesis.

In the current study, we leveraged a relatively large cohort of resected lung ADC precursors with available genomic and epigenetic profiling data to characterize the immune contexture across normal lung, AAH, AIS, MIA, and ADC by immune gene expression profiling, mIF, and TCR sequencing. Overall, there was a more suppressive and intensively regulated immune response, particularly T cell response, in later-stage lesions highlighting that the dynamic interaction between cancer cells and host immune surveillance has evolved toward immune escape along the pathogenesis of lung ADC. Notably, immune contexture progressed as a continuum from preneoplasia AAH to preinvasive AIS, to minimally invasive MIA and finally frankly invasive ADC. This is consistent with overall progressive genomic evolution from AAH to AIS, MIA, and ADC^7^ suggesting that early carcinogenesis of lung ADC is a gradual process shaped by host immune surveillance. This is in line with a similar study on the precursors of lung squamous cell carcinoma (SCC) by Mascaux and colleagues, which revealed the evidence of immune evasion along with the evolution of preinvasive SCC lesions^43^. Taken together, these data advocate for therapy targeting the immune microenvironment in patients with lung cancer precursors to prevent the development of invasive lung cancers. In light of these findings, therapeutic strategies reprogramming the tumor immune/inflammation microenvironment in patients with lung ADC precursors prior to intensive immunosuppression in invasive lung cancers may be beneficial. Interventions modifying the lung immune/inflammation microenvironment have long been proposed for lung cancer prevention^44^ and have shown promise in both preclinical models^45^ and patient cohorts^46^. Prospective lung cancer immunoprevention clinical trials such as randomized phase II of immunotherapy with Pembrolizumab for the prevention of lung cancer (IMPRINT-Lung; NCT03634241) testing immune checkpoint inhibitors (ICIs) in individuals with high-risk IPNs are currently underway to validate this hypothesis.

Identifying the molecular features associated with immune activation and evasion may facilitate establishing biomarkers for selection of patients who might benefit from immunoprevention. For example, *KRAS* mutations were correlated with more active immune response, while *EGFR* mutations were correlated with a cold immune microenvironment in this cohort of IPNs. It has been well established that *EGFR* mutations were associated with inferior response to immunotherapy in patients with advanced NSCLCs^47^. However, it is still unclear whether the impact of oncogenic mutations on the immune microenvironment and the response to immunotherapy is different in full-fledged cancers versus lung cancer precursors. Data from the IMPRINT-Lung trial, in which ICI is being tested in patients with high-risk IPNs regardless oncogenic driver status may shed light on this critical question and provide novel insights to guide future immunoprevention trials.

Overall, oncogene mutations, CNV burden, AI burden, and methylation status were all found to associate with the immune contexture in this cohort of IPNs, similar to advanced cancers^48,49^. However, the association with any single genomic or epigenetic feature was weak, suggesting heterogeneous but convergent evolution towards immune escape during early lung carcinogenesis. Our previous work suggested that early lung ADC carcinogenesis may predominantly follow the clonal sweep model, whereby certain subclones in early-stage preneoplasia turn into dominant clones in later-stage diseases while unfit subclones are eliminated^7^, primarily by the host immune system. In the early phases of carcinogenesis, stochastic genomic and epigenetic alterations lead to heterogeneous subclones with various combinations of molecular features that define the distinct biology of each subclone, including survival ability under selective immune pressure. Notably, the impact of genomic and epigenetic alterations on immune response are intertwined. For example, methylation may directly affect immune response by regulating the expression of immune genes^50^ and potential neoantigens^51^ or indirectly by increasing DNA vulnerability for development of CNV and somatic mutations that can subsequently influence the immune microenvironment. Global hypomethylation is known to associate with CIN^52^ and increased rate of somatic mutations^53^ and indeed, global methylation level was negatively associated with TMB and CNV burden in this cohort of lesions^8^. Since high CNV burden is associated with a cold immune microenvironment^29^ while high TMB can increase tumor immunogenicity and facilitate immune recognition and elimination of cancer cells^54^, the impact of global hypomethylation (associated with both high CNV burden and high TMB) on anti-tumor immunity is complicated. Eventually, the outgrowth of subclones is determined by the accumulated effects of all molecular aberrations. Only the cells with the combination of molecular features enabling their rapid proliferation and escape from immune attack will survive and outgrow into the dominant clones in invasive cancers. For example, though advanced IPNs tend to harbor a high TMB that could lead to active anti-tumor immune response, they also exhibited higher CNV burden, higher incident of HLA LOH and decreased global methylation, all of which are associated with a cold tumor immune microenvironment, resulting in an overall cold immune microenvironment in advanced IPNs.

To the best of our knowledge, this is the first systemic study on immune landscape and associated molecular features in IPNs of different histologic stages during early lung adenomatous carcinogenesis. However, this study has several technical and scientific limitations. From a technical aspect, our immune profiling lacked high-resolution T cell subtyping because of limited numbers of markers assessed by mIF and immune genes profiled. Additionally, the scarcity of resected IPNs and small sizes of these IPNs hindered more comprehensive molecular profiling. Although methylation data suggested that promoter hypermethylation could lead to neoantigen depletion and immune escape, these findings remain speculative in absence of transcriptomic analysis of predicted neoantigens. Moreover, due to technical challenges with antibodies, we were unable to directly assess CD4 expression by mIF. Numerous studies have demonstrated that double-negative (CD3+CD4−CD8−) and double-positive (CD3+CD4+CD8+) T cells only constitute very small portions of T lymphocytes^55–59^ suggesting using CD3+CD8− to infer CD3+CD4+ cells could likely represent these cells accurately, though it still remains suboptimal. With the emergence of advanced technologies which combine larger numbers of markers (i.e. imaging mass cytometry^20^) the interaction of T cell subtypes could be assessed at a more granular level in these scarce specimens.

Beyond the challenges outlined above, unveiling stepwise molecular evolution associated with the progression from preneoplasia to invasive ADC is scientifically difficult due to the uncertain clinical course and biological complexity of these lesions. First, in the current study and the majority of previous studies regarding this subject, all molecular and immune data were derived from resected IPNs, which offered a single molecular snapshot of the evolutionary process of lung ADCs. Although a linear model from AAH to AIS, MIA and invasive ADC has been proposed for lung adenomatous carcinogenesis, whether all AAH/AIS/MIA eventually progress to invasive lung ADC and whether all invasive ADC follows this linear evolutionary trajectory is unknown. Second, intratumor heterogeneity of preneoplastic/cancer cells and immune cells present not only in full-fledged lung cancers^26,60–62^, but also in their precursors^7,63^, further increases the difficulty in identifying the molecular events that drive neoplastic evolution. Third, all patients in this study were from Japan and China; whether these findings are broadly applicable in Western populations remains unknown. In the face of such complexities, international collaborative studies on larger cohorts of IPNs with long-term follow up and longitudinal biopsies are needed to dissect the evolutionary trajectory of early lung adenomatous carcinogenesis and its underlying molecular mechanisms. Finally, these intriguing findings are based on association rather than direct causation. Therefore, functional studies using model systems including human relevant animal models are warranted to understand the actual molecular mechanisms underlying early lung adenomatous carcinogenesis.

## Methods

### Patients and tissue processing

Specimens were collected from 53 patients presenting with pulmonary nodules, who underwent surgical resection at Nagasaki Hospital (Japan) or Zhejiang Cancer Hospital (China) from 2014 to 2017 as described previously^7^. Written informed consent was obtained from all patients. The study was approved by the Institutional Review Boards (IRB) at MD Anderson Cancer Center, Nagasaki University Graduate School of Biomedical Sciences, and Zhejiang Cancer Hospital. Hematoxylin and eosin (H&E) slides of each case were reviewed independently by two lung cancer pathologists to confirm the diagnosis.

### DNA and RNA extraction

After pathologic assessment, macro-dissection was performed to obtain premalignant cells or tumor cells for DNA and RNA extraction. DNA and RNA samples were isolated using the AllPrep® DNA/RNA FFPE Kit (Qiagen, Hilden, Germany). DNA samples were quantified by NanoDrop 1000 Spectrophotometer (Thermo Scientific, Wilmington, DE, USA) and RNA samples were quantified using RNA High sensitivity kit on the Qubit 3.0 fluorometer (Thermo Fisher Scientific, USA). RNA quality was evaluated with RNA integrity number (RIN)^64^ using RNA ScreenTape assay in Agilent 4200 TapeStation system (Agilent Technologies, USA).

### Gene expression profiling of immune-related genes

The nCounter® PanCancer Immune Profiling Panel (NanoString Technologies, Inc., Seattle, WA, USA), which contains 770 genes (including 730 immune-related genes and 40 housekeeping genes), was applied for gene expression profiling as previously described^65,66^ and nSolver 4.0 software (NanoString Technologies) was applied for quality control with default setting, and all samples passed QC check. Background correction was performed with negative controls, followed by two-steps normalization using geometric mean of 6 positive controls and 40 housekeeping genes. Then global adjustment was achieved by quantile-normalization to remove any unwanted technical variations, which was followed by log2 transformation to obtain variance stabilization. Adequate processing was assessed by MA plots of tumor-normal pairs. One-way ANOVA test was applied to identify differentially expressed genes (DEGs) in different stages. We modeled the p-values using a beta-uniform mixture (BUM) model, combined with false discovery rate (FDR < 0.1%) to determine a cutoff for *p*-values^67^. Ingenuity Pathway Analysis (IPA) software (Quiagen, Hilden, Germany)^68^ was applied to identify pathways enriched by these DEGs, with Core Analysis based on human species and lung tissue. IPA identifies the top canonical pathways enriched by these DEGs with a right-tailed Fisher’s exact test and calculates a *z*-score to predict activation status of each pathway by comparing input genes and the scored activation pattern. TIMER was applied to infer the infiltration of various immune cell subtypes by de-convolution of gene expression data^69^.

### Multiplex immunofluorescence staining and multispectral analysis

The multiplex immunofluorescence (mIF) analysis was applied only for samples with good quality based on H&E staining of the same specimen. The mIF staining was performed on unstained slides of FFPE specimens using the Opal 7-Color fIHC Kit (Akoya Biosciences, USA) as previously described^70^. Eight immune markers were placed in two 6-antibody mIF panel as followed: panel 1 contained pancytokeratin (AE1/AE3; epithelial marker; dilution 1:300; Dako, Carpinteria, CA), PD-L1 (clone E1L3N, dilution 1:100; Cell Signaling Technology, Beverly, MA), PD1 (clone EPR4877-2, dilution 1:250; Abcam, Cambridge, MA), CD3 (T lymphocyte marker; dilution 1:100; Dako), CD8 (cytotoxic T cell marker; clone C8/144B, dilution 1:20; Thermo Fisher Scientific, Waltham, MA), and CD68 (macrophage marker; clone PG-M1, dilution 1:450; Dako); and panel 2 contained pancytokeratin, CD3, CD8, CD45RO (memory T cell marker; clone UCHL1, ready to use; Leica Biosystems, Buffalo Grove, IL), Granzyme B (cytotoxic lymphocyte marker; clone F1, ready to use; Leica Biosystems), and FoxP3 (regulatory T cell marker; clone 206D, dilution 1:50; BioLegend, SanDiego, CA). Human tonsil FFPE tissues were included and used as positive and negative (autofluorescence) control with the primary antibodies plus fluorophores and with primary antibodies without fluorophores, respectively. The stained slides were scanned using Vectra 3.0 multispectral microscope system (Akoya Biosciences, USA) under fluorescent illumination as previously described^71^, first in low magnification (×10), then the representative regions of interest (ROIs) were selected with Phenochart1.0.9 viewer (Akoya Biosciences, USA) and finally scanned in high magnification (×20). The ROIs (669 × 500 μm each) for mIF analysis were then selected after comparing with H&E slides to capture malignant and premalignant cell cluster and various elements of heterogeneity. The corresponding normal ROIs were selected in the farthest field of tumor periphery with morphological normal tissue on the same slide. Each ROI with panel 1 and panel 2 was overlapped with sequential sections. The target areas were analyzed by in Form 2.4.4 software (Akoya Biosciences, USA). Next, the ROI was divided into two compartments: epithelial compartment (alveolar wall or malignant cell nests) and alveolar air space or tumor stroma compartment. The individual cells were recognized by DAPI nuclei staining and co-localization markers, their represented immune subtypes in panel 1 and panel 2 were summarized in Supplementary Data 3. Percent data are calculated by dividing the total nucleated cells on each panel for each sample, and are used for further analysis.

### Profiling of TCRβ repertories

Immunosequencing of the CDR3 regions of human TCRβ chains was performed using ImmunoSeq (hsTCRβ Kit, Adaptive Biotechnologies) and T cell clonality and diversity were calculated as described previously^26,37^. Briefly, T cell clonality represents a metric of T cell proliferation and reactivity, and it is defined as 1-Pielou’s evenness and calculated based on productive rearrangements by $1+\frac{{\sum }_{i}^{N}{\mathrm{P}}{\mathrm{i}}{\mathrm{log}}2({\mathrm{P}}{\mathrm{i}})}{\log \,2(N)}$, Where pi is the proportional abundance of rearrangement *i*, and *N* is the total number of rearrangements. Clonality ranges from 0 to 1, values approaching 0 indicate completely evenly distributed frequency of different clones (subclonal), whereas values approaching 1 indicate distinct asymmetric distribution in which only a few activated clones present at high frequencies (monoclonal). We applied Inverse Simpson index to derive T cell diversity, the sum over all observed rearrangements of the square fractional abundances of each rearrangements using productive templates $\frac{1}{{\sum }_{i=1}^{S}{\mathrm{P}}{{\mathrm{i}}}^{2}}$, where pi is the proportional abundance of rearrangement *i*, and *S* is the total number of rearrangements. Inverse Simpson ranges from 1 to infinite, where a sample with little variation or abundance has a value approaching 1, and a sample with maximally diversity and evenly distribution has a value approaching infinite. T cell density, an estimate of T cell infiltration in the tumor, was calculated by normalizing TCR-β template counts to the total amount of DNA usable for TCR sequencing, where the amount of usable DNA was determined by PCR-amplification and sequencing of housekeeping genes expected to be present in all nucleated cells.

### Detection of allele-specific HLA loss

Loss of heterogeneity of human leukocyte antigen (HLA) was assessed as previously descried^31^. Briefly, class I HLA alleles for each HLA gene was inferred by POLYSOLVER using a two-step Bayesian classification approach, which takes into account the base qualities of aligned reads, observed insert sizes, as well as the ethnicity-dependent prior probabilities of each allele. Tumor purity and ploidy were estimated using ASCAT. Then Loss Of Heterozygosity in Human Leukocyte Antigen (LOHHLA) algorithm was applied to detect allele-specific HLA loss in each sample. Briefly, logR and BAF across each HLA gene loci was obtained by binning the coverage at mismatch positions between homologous HLA alleles, and HLA haplotype specific copy numbers were then calculated based on logR and BAF values from the corresponding bin adjusted by tumor purity and ploidy. The median value of binned allelic copy number was used to determine LOH, where a copy number of <0.5 indicated allele loss and LOH was determined if *p* < 0.01.

### Prediction of neoantigens

All the peptides with 9–12 amino acids that spanning missense or stop gain mutations were extracted as mutant peptides, to identify candidate peptides binding to MHC Class I or II molecules. The affinity of 9–12 peptides binding to MHC Class I molecules were predicted using the NetMHCPan3.0 binding algorithm^72^. The affinity of 9–12 mer peptides binding to MHC Class II molecules were predicted using the NetMHCIIPan3.1 binding algorithm^73^. The threshold for binding peptides is defined as half-maximum inhibitory concentration affinity value (IC50) < 500 nM or a rank percentage score< 2%. HLA-A, HLA-B, HLA-C are included in MHC class I molecules, and HLA-DR, HLA-DP and HLA-DQ are included in MHC Class II molecules.

### Integration of genomic and methylation profiling data

Somatic mutations, allelic imbalance (AI), copy number variations (CNV), oncogene mutations, and global methylation analysis in the same cohort were performed in previous studies^7,8^.

### Statistical analysis

Different statistical models were applied to assess the association among immune data, genomic data, and methylation data. For association between two continuous variables, spearman’s rank correlation test was used. For association between one continuous variable and one categorical variable, Wilcoxon rank-sum test (categorical variable with two levels) and Kruskal–Wallis H test (categorical variable with more than two levels) were applied. The FDR method was used for multiple testing adjustment of *p*-values^74^. All *p*-values are calculated with two-sided test, and *p* < 0.05 was considered to be statistically significant.

### Reporting summary

Further information on research design is available in the Nature Research Reporting Summary linked to this article.

## Supplementary information

Supplementary Information

Description of Additional Supplementary Files

Supplementary data

Reporting Summary

## Data Availability

The data for WES has been deposited at European Genome-phenome Archive (EGA), under the accession code: EGAS00001004960 and the data for RRBS is under EGAS00001004610. Both WES and RRBS datasets are available under restricted access, which can be obtained by contacting Jianjun Zhang (JZhang20@mdanderson.org). The gene expression data of Nanostring nCounter is deposited to GEO under https://www.ncbi.nlm.nih.gov/geo/query/acc.cgi?acc=GSE169033. TCRseq dataset is available in immuneACCESS under https://clients.adaptivebiotech.com/pub/dejima-2021-nc. GSE102511 was retrieved from GEO. Source data is provided as a source data file. All other data may be found within the main manuscript and supplementary information or available from the authors upon request.

## Source data

Source Data
