# Supplementary material for: Immune evolution from preneoplasia to invasive lung adenocarcinomas and underlying molecular features
Source: Nat Commun. 2021 May 11;12:2722. doi: 10.1038/s41467-021-22890-x (PMC8113327; doi:10.1038/s41467-021-22890-x)
Supplement: Supplementary file 1 — Supplementary Information [file 41467_2021_22890_MOESM1_ESM.pdf]

Supplementary Figure 1

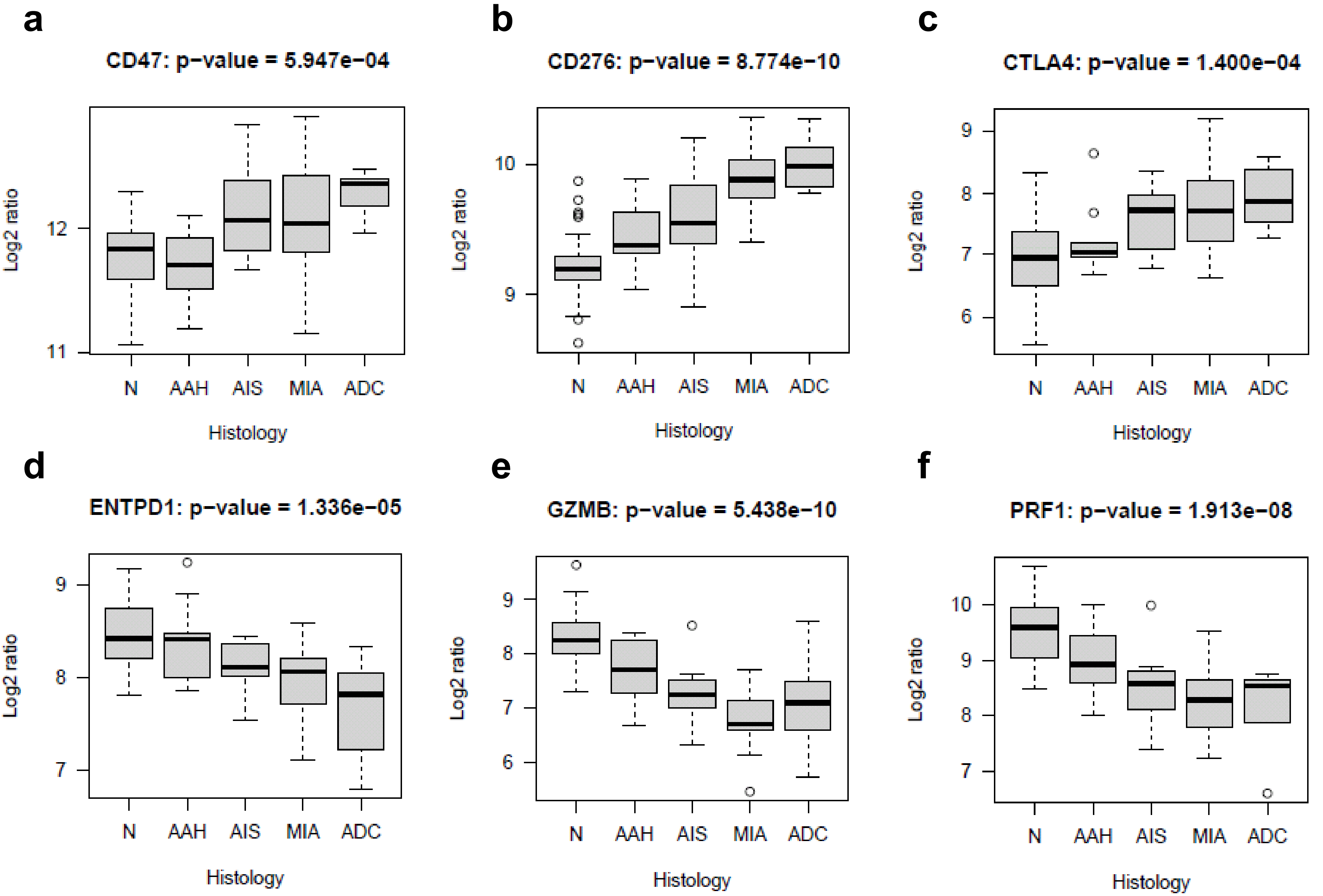

Supplementary Figure 1. Example genes differentially expressed from preneoplasia to invasive lung adenocarcinoma.

Gene expression of (a) CD47, (b) CD276, (c) CTLA4, (d) ENTPD1, (e) GZMB and (f) PRF1 was performed using the nCounter PanCancer Immune Profiling Panel (Nanostring). The difference in expression of each gene among different stages was evaluated using two-sided Kruskal-Wallis H test. The crossbars represent the median, and the bounds of the boxes represent the lower (Q1) and upper (Q3) quartiles of log2 transformed gene expression values, the whiskers represent the most extreme data points which are no more than 1.5*IQR (Inter-Quantile Range). The data falling outside of the whiskers are plotted as the outliers. n = independent IPNs for 38 normal lung (NL), 9 atypical adenomatous hyperplasia (AAH), 11 adenocarcinoma in situ (AIS), 21 minimally invasive adenocarcinoma (MIA), 6 invasive adenocarcinoma (ADC). (Source data is provided as a source data file.)

Supplementary Figure 2

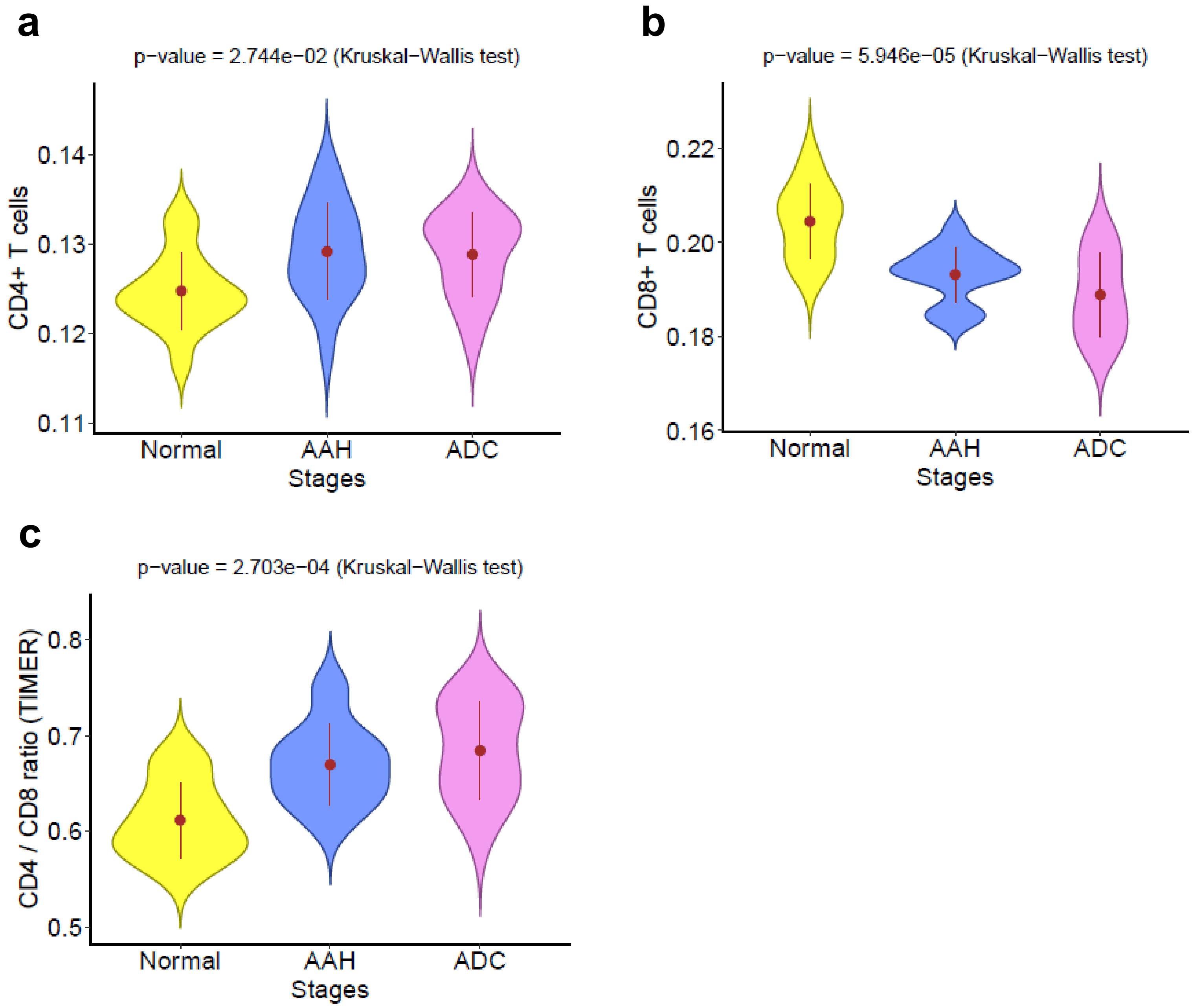

Supplementary Figure 2. Progressive changes of immune cell infiltration in normal lung, preneoplasia and invasive lung adenocarcinoma.

Immune cell fractions including (a) CD4+ T cells, (b) CD8+ T cells, (c) CD4 /CD8 ratio were estimated using TIMER based on previously published RNA sequencing data from an independent cohort (GSE102511). Error bars indicate 95% confidence intervals and solid point represent mean value in each stage. The difference of cell fraction among stages was evaluated using two-sided Kruskal-Wallis H test. Data from 17 patients was used including NL: Normal lung tissue, AAH: Atypical adenomatous hyperplasia, ADC: Invasive adenocarcinoma. (Source data is provided as a source data file.)

Supplementary Figure 3

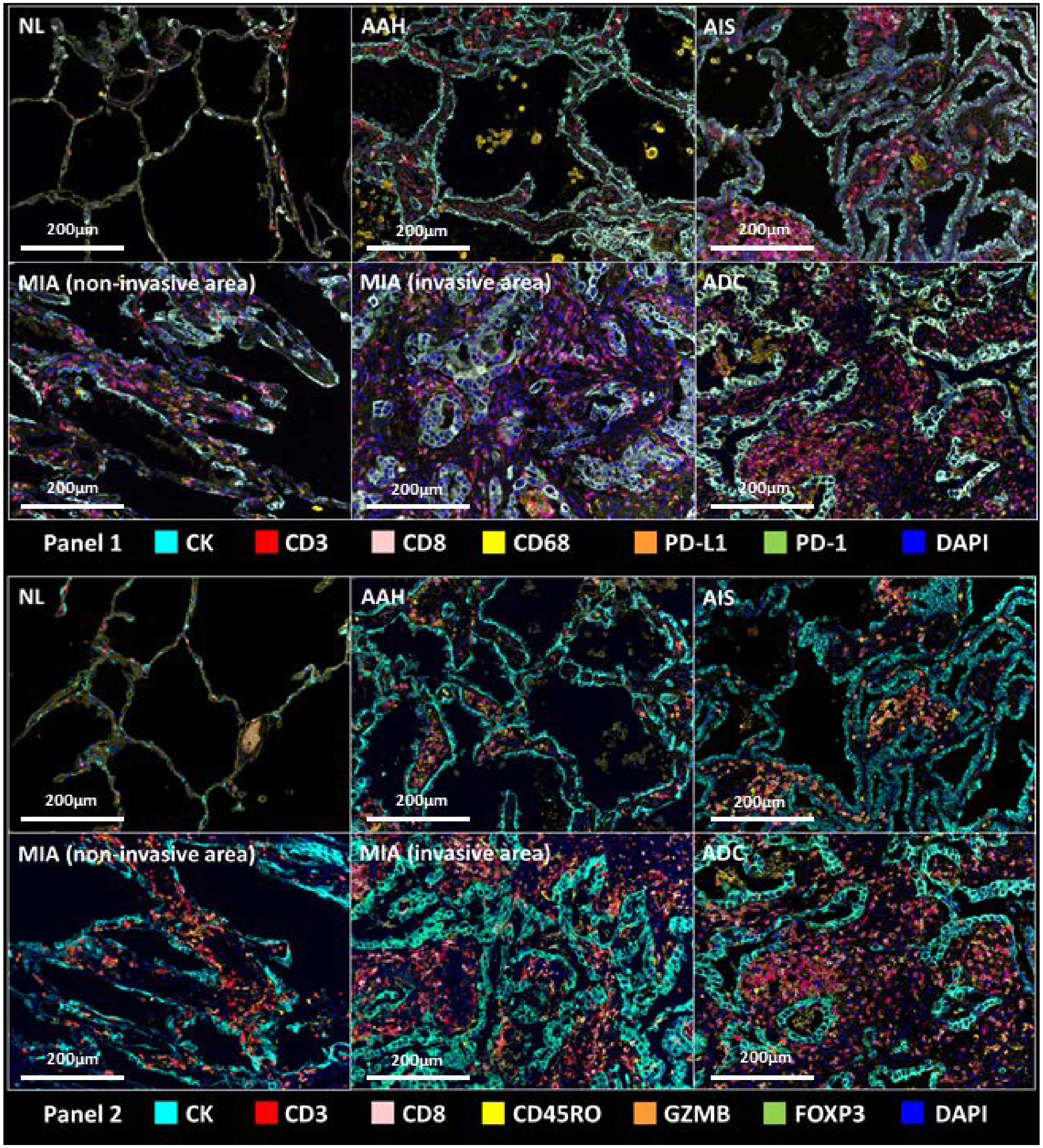

Supplementary Figure 3. Representative multiplex immunofluorescence (mIF) images in IPNs of different histologic stages.
Microphotographs of representative patient including normal, AAH, AIS, MIA and ADC from multiplex IF (×20 magnification), scale bars represent 200μm on each image (669 μm×500μm). Immune panel 1: CK, CD3, CD8, CD68, DAPI. Immune panel 2: CK, CD3, CD8, CD45RO, GZMB, FOXP3, DAPI. Data from 37 patients was used in mIF. (Source data is provided as a source data file.)

Supplementary Figure 4

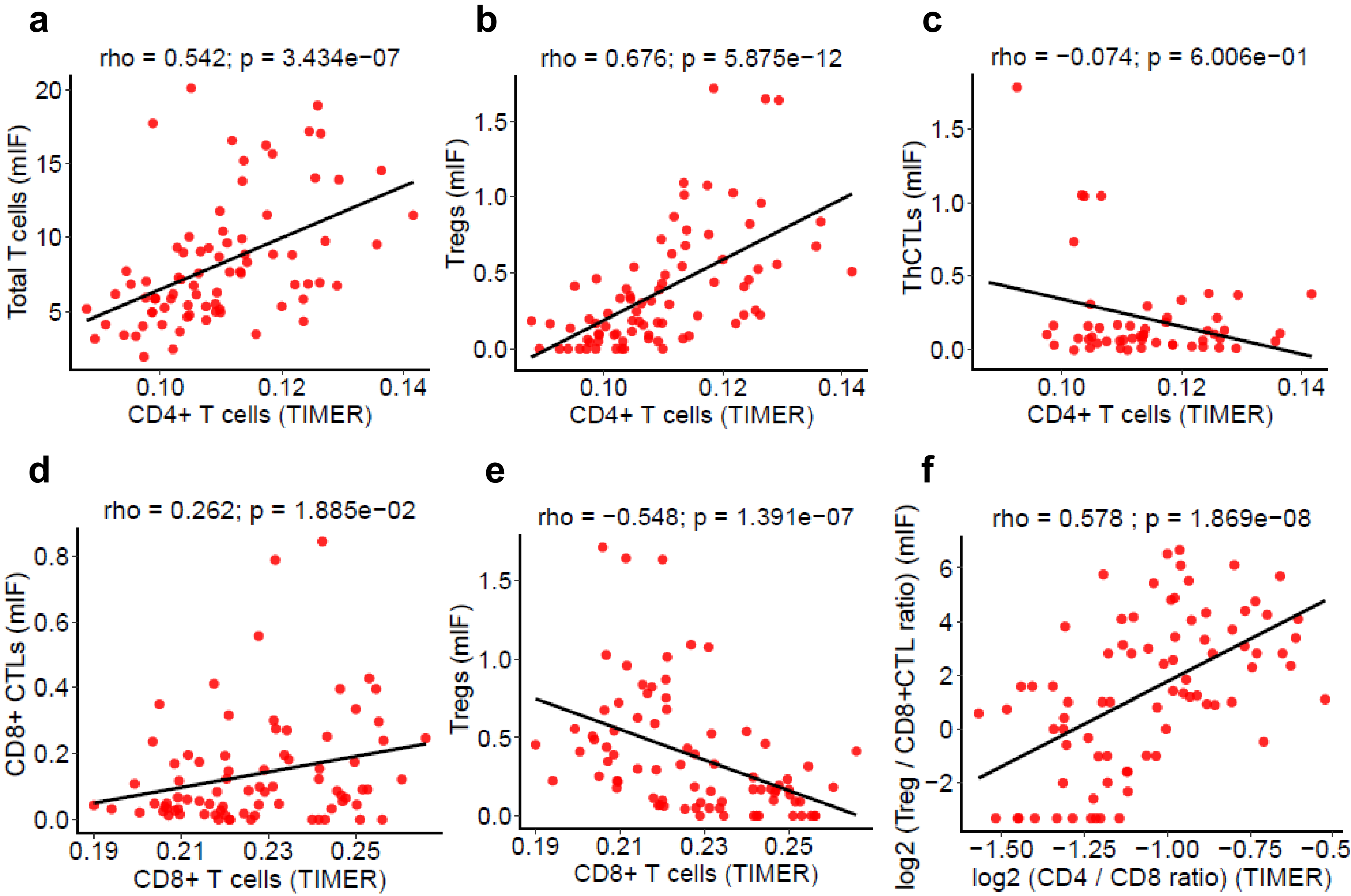

Supplementary Figure 4. The correlation of CD4+/CD8+ T cells inferred from immune gene expression with T cell subtypes defined by mIF.

Fractions of (a-c) CD4+ T cells , (d, e) CD8+ T cells and (f) CD4/CD8 ratio estimated using TIMER based on the gene expression from nCounter PanCancer Immune Profiling Panel (x-axis) were correlated to (a) total T cells (CD3+), (b, e) regulatory T cell (CD3+CD8-FoxP3+), (c) ThCTLs (CD3+CD8-granzyme B+), (d) CD8+CTLs (CD3+CD8+granzymeB+), and (f) Treg/CD8+CTL ratio (log2 transformed) measured by mIF (y-axis). The correlation coefficient (rho) was assessed by two-tailed Spearman's rank correlation test. Data from 37 patients was used in correlation analysis. (Source data is provided as a source data file.)

Supplementary Figure 5

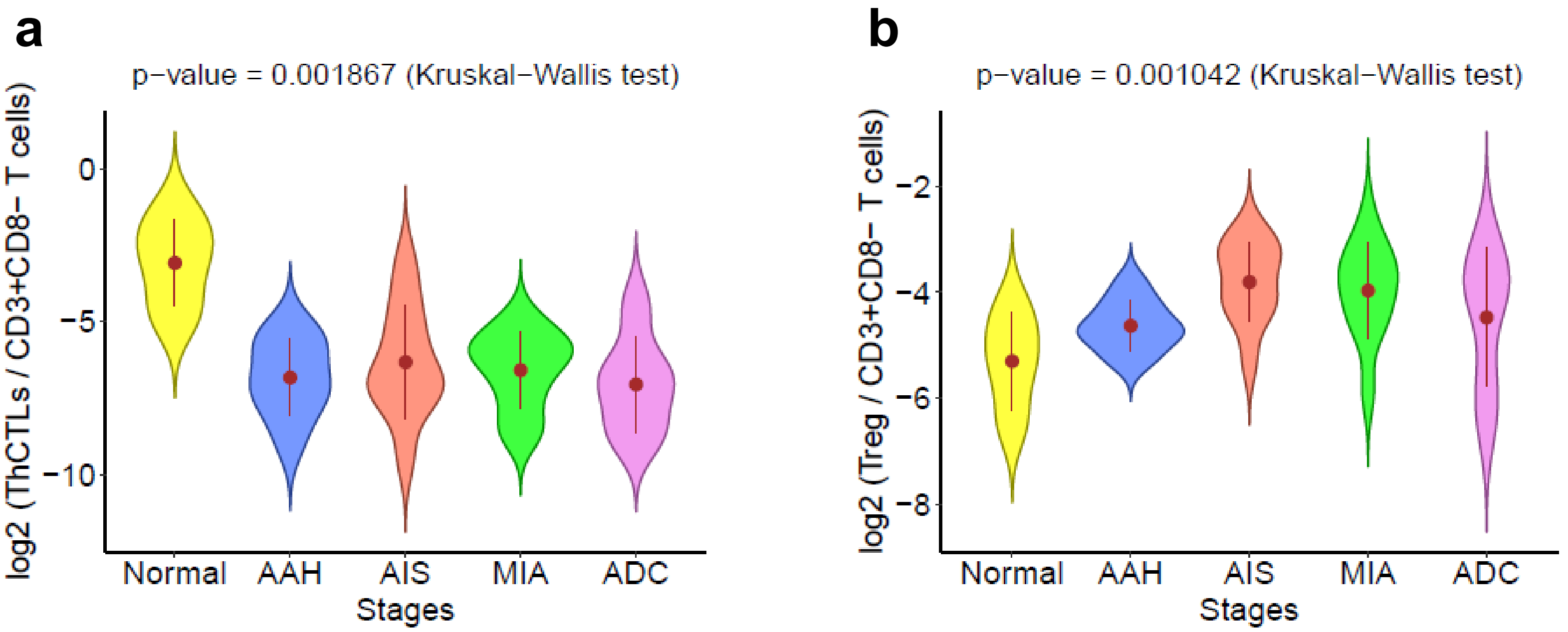

Supplementary Figure 5. The proportion of CD3+CD8- T cell subtypes defined by mIF in IPNs of different stages.
The proportion of (a) ThCTLs (CD3+CD8-GZMB+) and (b) Tregs (CD3+CD8-FOXP3+) among CD3+CD8- T cells. The solid dots represent the mean values in each stage with the 95% confidence intervals as error bars. The difference in cell fractions among different stages was evaluated using two-sided Kruskal-Wallis H test. The ratio were log2 transformed for visualization. Data from 37 patients was used in mIF. (Source data is provided as a source data file.)

Supplementary Figure 6

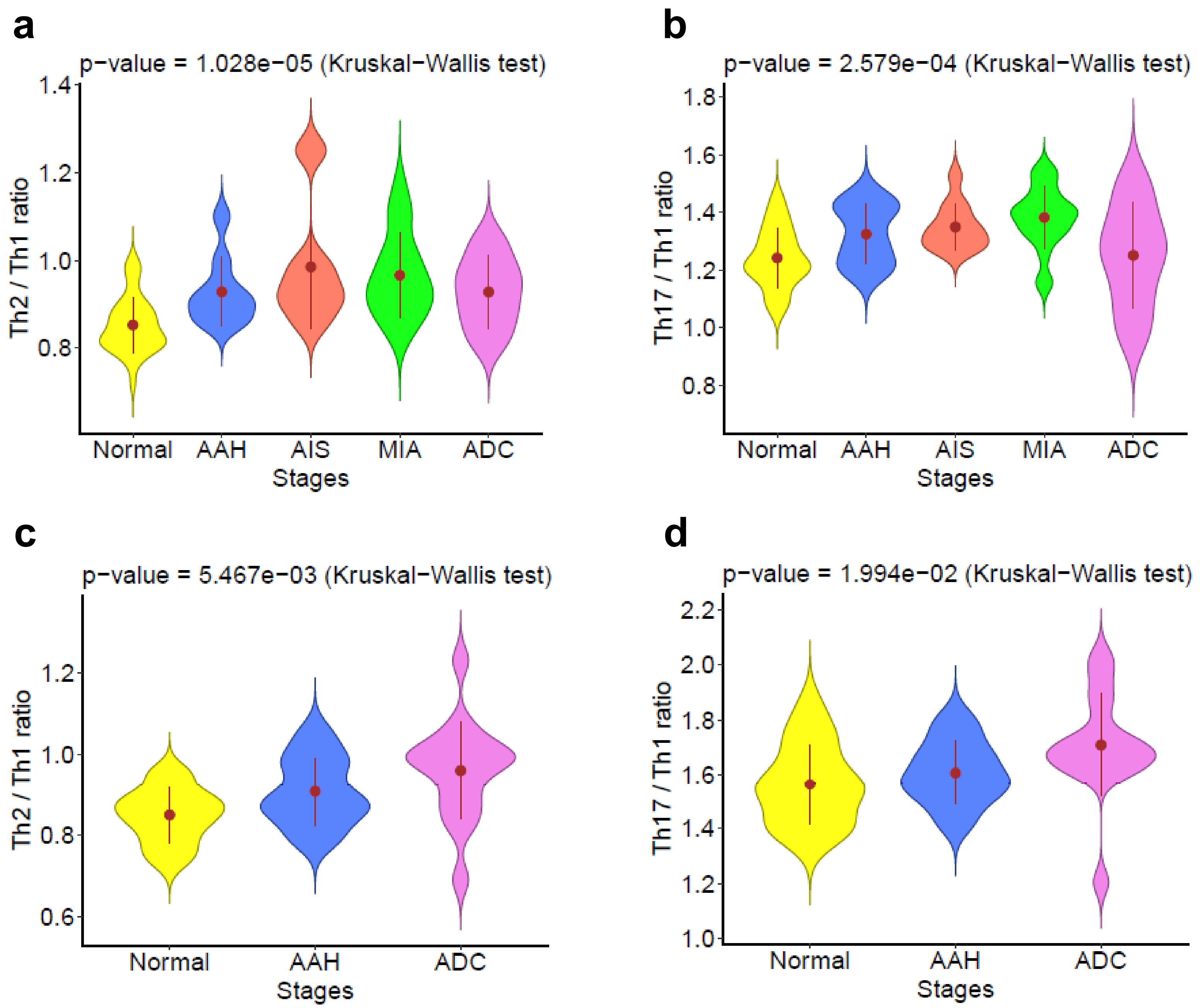

Supplementary Figure 6. The ratio of gene expression signatures of main T helper subtypes. The ratio of (a) Th2/Th1 and (b) Th17/Th1 in IPNs of different stages from the current cohort with 38 patients. The ratio of (c) Th2/Th1 and (d) Th17/Th1 in normal lung, AAH and invasive ADC from a previously published independent cohort with 17 patients (GSE102511). Th1 markers: IFNG, IL12A, IL12B; Th2 markers: IL4, IL5, IL13; Th17 markers: IL17A, IL17F, IL23A, IL6, TGFB1, TGFB2. Solid dots represent the means of marker gene expression in all IPNs of each stage with 95% confidence intervals as error bars. The difference between different stages was assessed by two-sided Kruskal-Willis H test. (Source data is provided as a source data file.)

Supplementary Figure 7

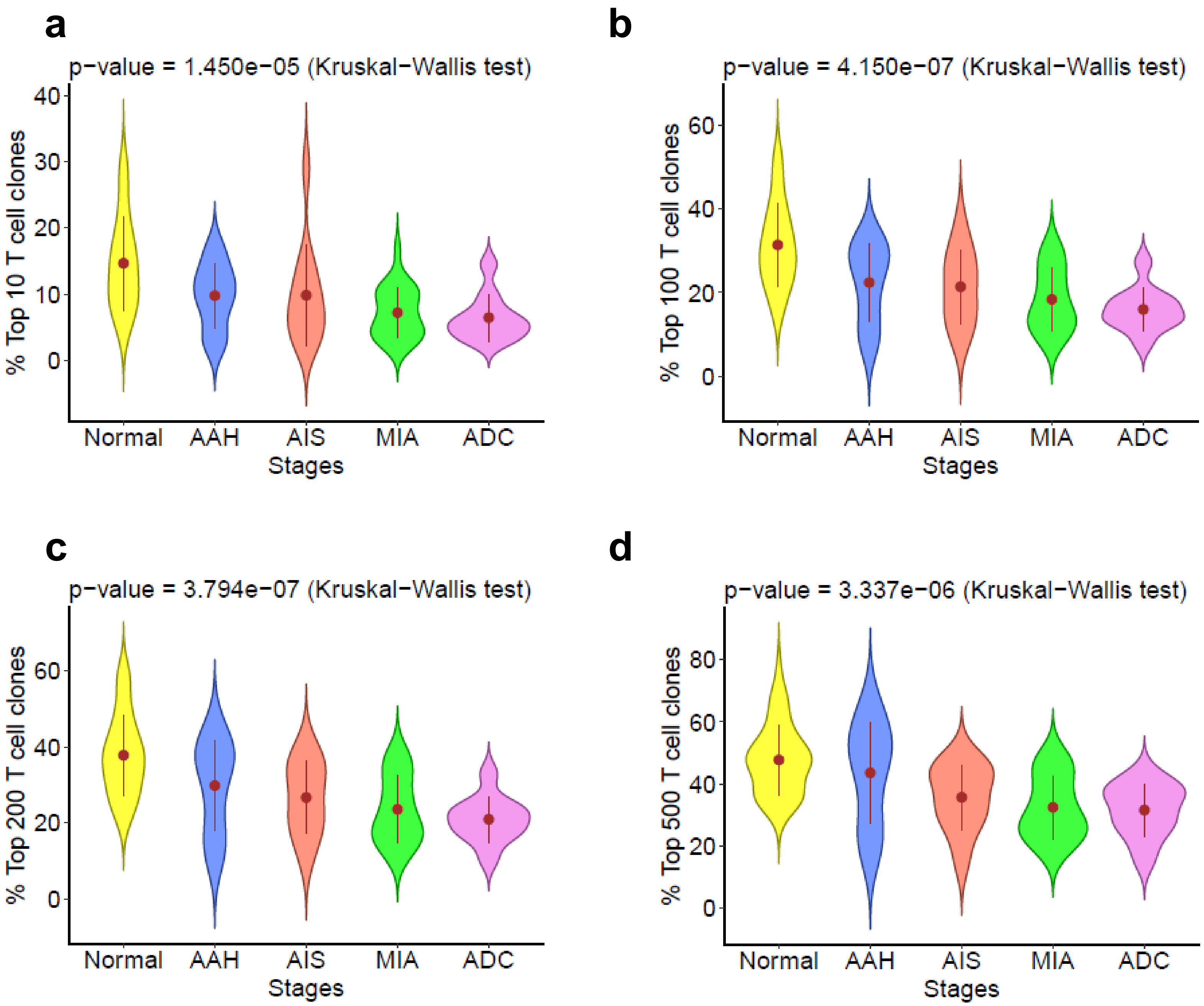

Supplementary Figure 7. The top T cell clone frequencies in IPNs of different stages. Frequencies of (a) top 10, (b) top 100, (c) top 200 and (d) top 500 T cell clones in the Normal (yellow), AAH (blue), AIS (red), MIA (green) and ADC (purple). Solid dots represent the means of top T cell clone frequencies in all IPNs of each stage with 95% confidence intervals as error bars. The difference between different stages were assessed by two-sided Kruskal-Wallis H test. Data from 51 patients was used in TCRseq. (Source data is provided as a source data file.)

Supplementary Figure 8

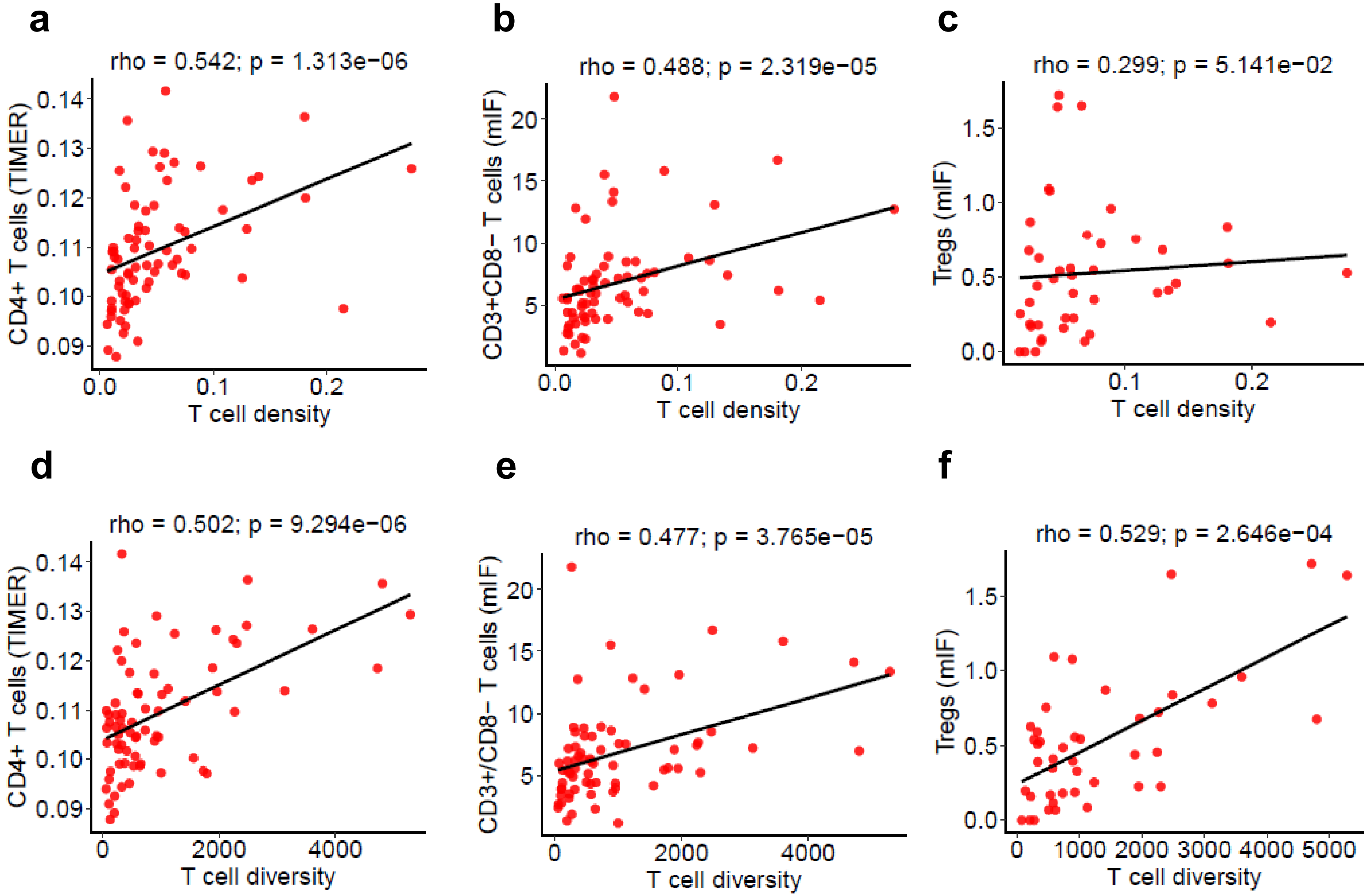

Supplementary Figure 8. The association between TCR density/diversity and T cell subtypes.
The association of T cell density from TCR sequencing with (a) CD4+ T cells inferred from gene expression profiling using TIMER, (b) CD3+CD8- T cells and (c) Tregs (CD3+CD8-FOXP3) measured by mIF.
The association of T cell diversity from TCR sequencing with (d) CD4+ T cells inferred from gene expression profiling using TIMER, (e) CD3+CD8- T cells and (f) Tregs (CD3+CD8-FOXP3) measured by mIF.
The correlation coefficient (rho) was assessed by two-tailed Spearman's rank correlation test. Data from 35 patients was used in correlation analysis. (Source data is provided as a source data file.)

Supplementary Figure 9

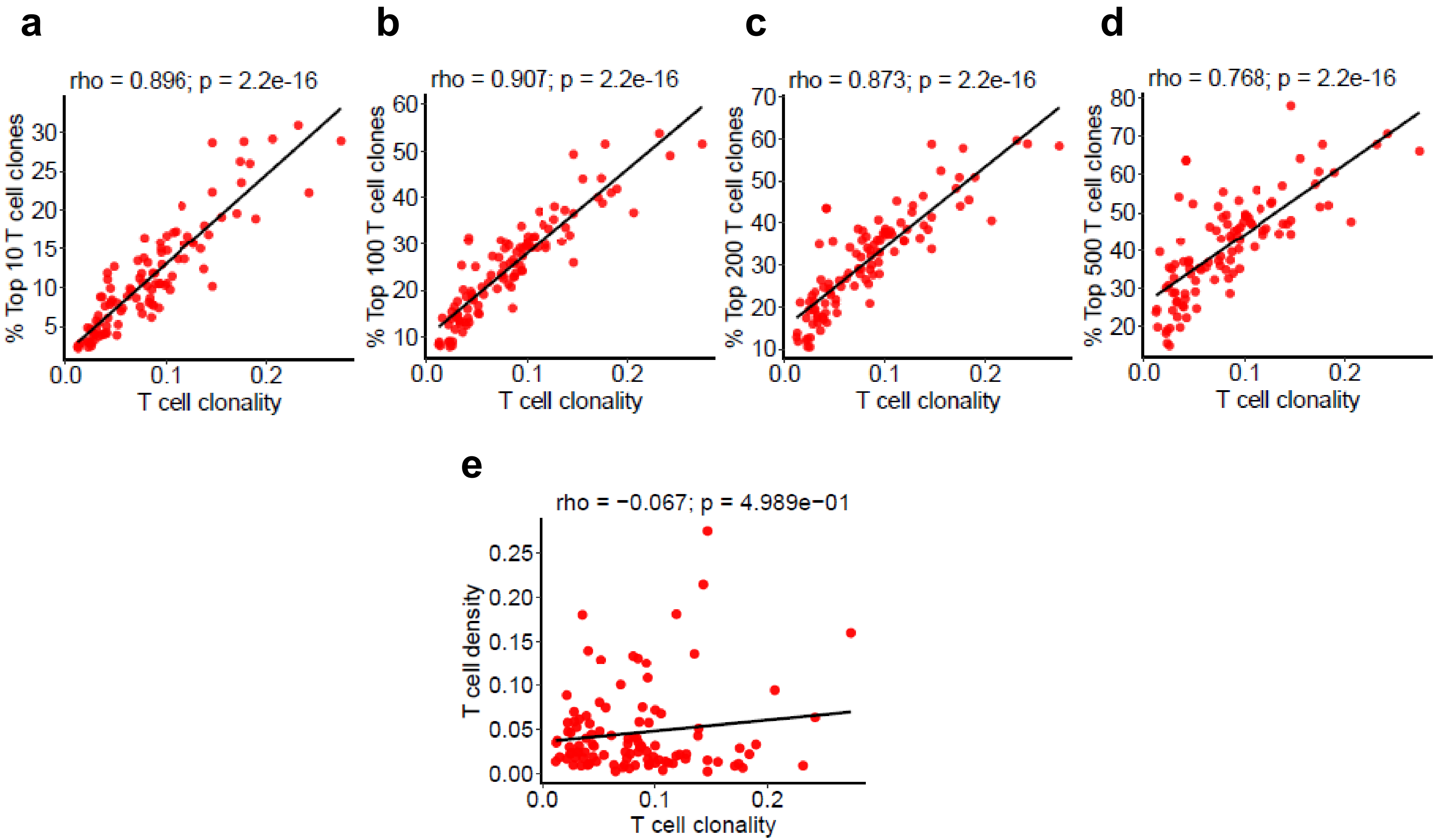

Supplementary Figure 9. The association between T cell clonality and the frequency of top T cell clones. The y-axis represents the sum frequencies of T cells from the (a) Top 10 clone, (b) Top 100 clones, (c) Top 200 clones, (d) Top 500 clones in each IPN measured by TCR sequencing. (e) The association between T cell clonality and T cell density. The correlation coefficient (rho) was assessed by two-tailed Spearman's rank correlation test. Data from 51 patients was used in TCRseq. (Source data is provided as a source data file.)

Supplemental Figure 10

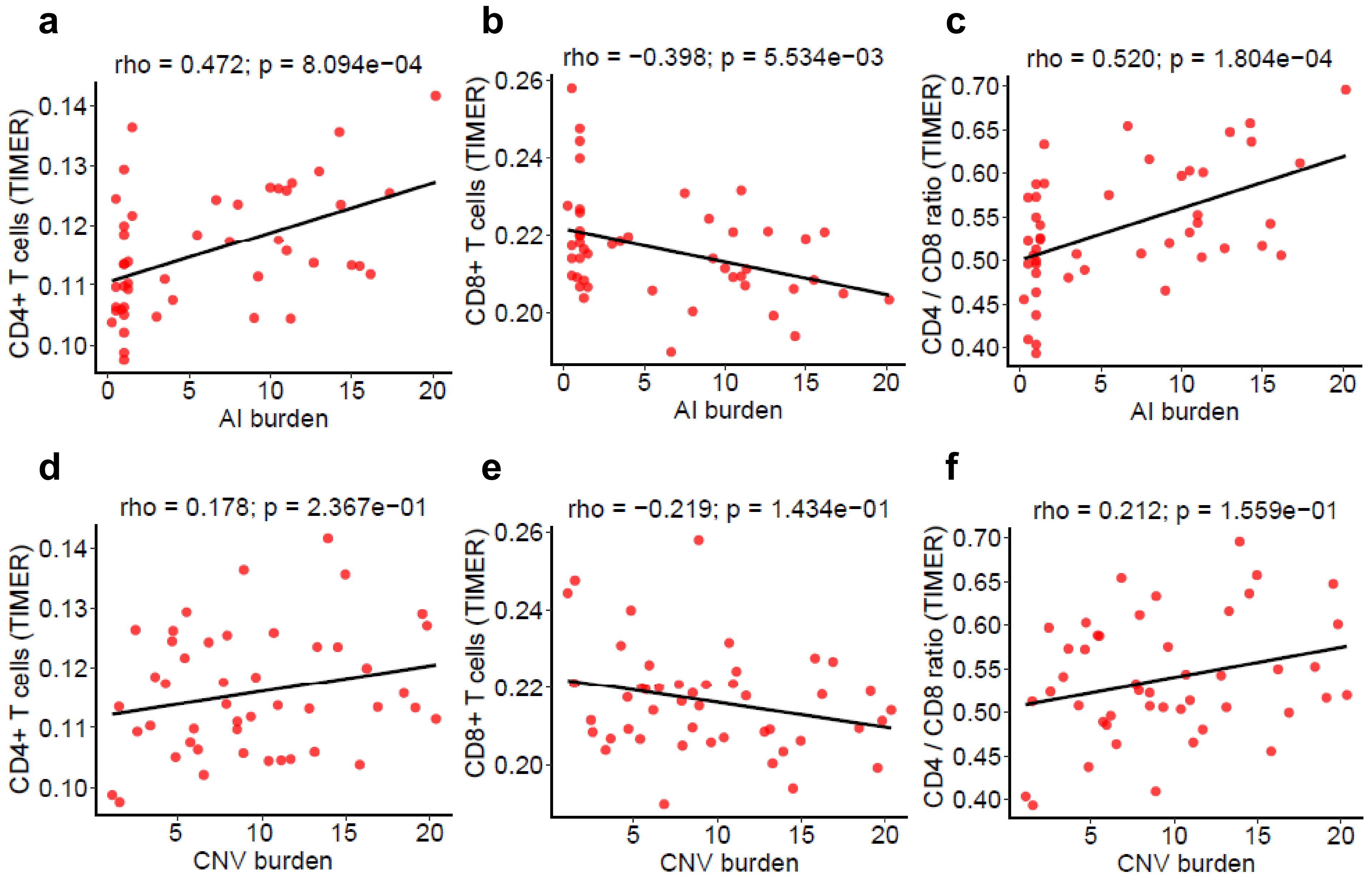

Supplementary Figure 10. The potential impact of chromosomal copy number changes on immune infiltration. The correlation between AI burden (number of AI events) and infiltration of (a) CD4+ T cells, (b) CD8+ T cells, (c) CD4/CD8 ratio inferred from gene expression by TIMER. The correlation between CNV burden (normalized as the number of genes with CNV) and infiltration of (d) CD4+ T cells, (e) CD8+ T cells, (f) CD4/CD8 ratio inferred from gene expression by TIMER. The correlation coefficient (rho) was assessed by two-tailed Spearman's rank correlation test. Data from 38 patients was used in correlation analysis. (Source data is provided as a source data file.)

Supplementary Figure 11

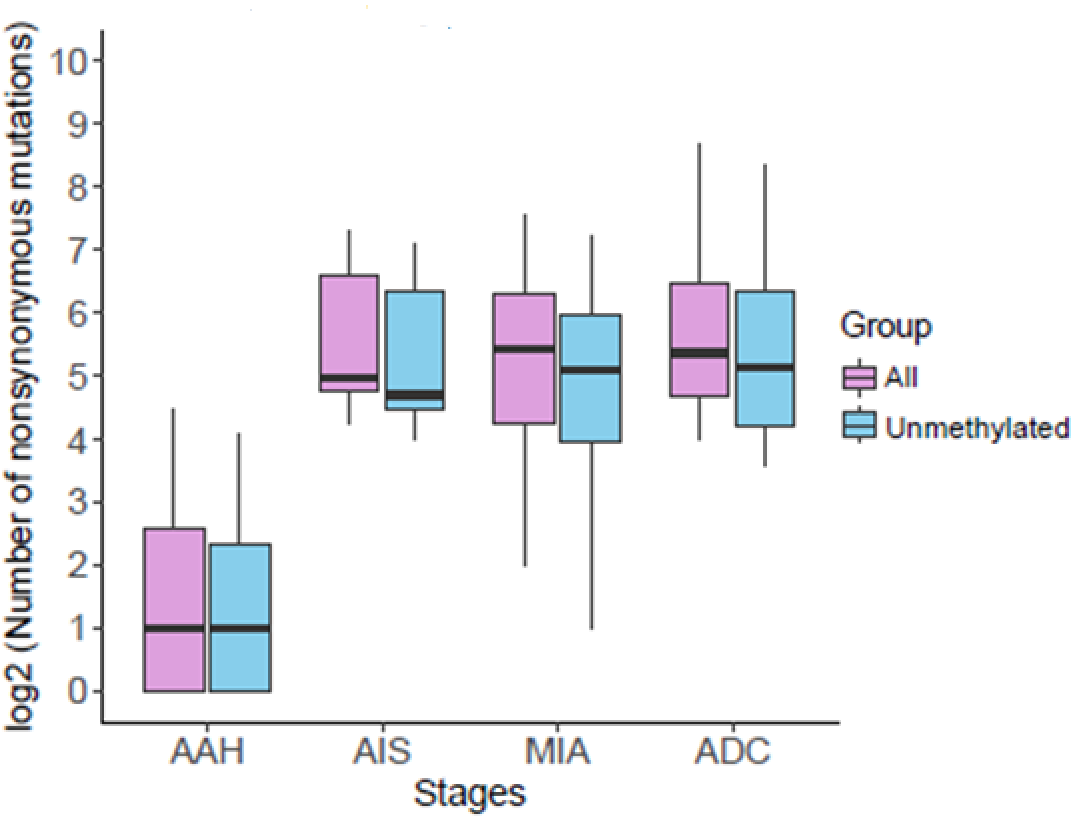

Supplementary Figure 11. Promoter hypermethylation and mutation burden from preneoplasia to invasive lung adenocarcinoma. The number of all nonsynonymous mutations in each histologic stage is shown as purple boxes and the number of nonsynonymous mutations from genes without promoter methylation (<30% CpG sites methylated) is shown as blue boxes. The crossbars represent the median for the number of nonsynonymous mutations, and the bounds of the box represent the lower (Q1) and upper (Q3) quartiles, the whiskers represent the most extreme data points which are no more than 1.5*IQR (Inter-Quantile Range), the outliers are not shown. n = independent IPNs for 13 AAH, 12 AIS, 18 MIA, 11 ADC. (Source data is provided as a source data file.)

Supplementary Figure 12

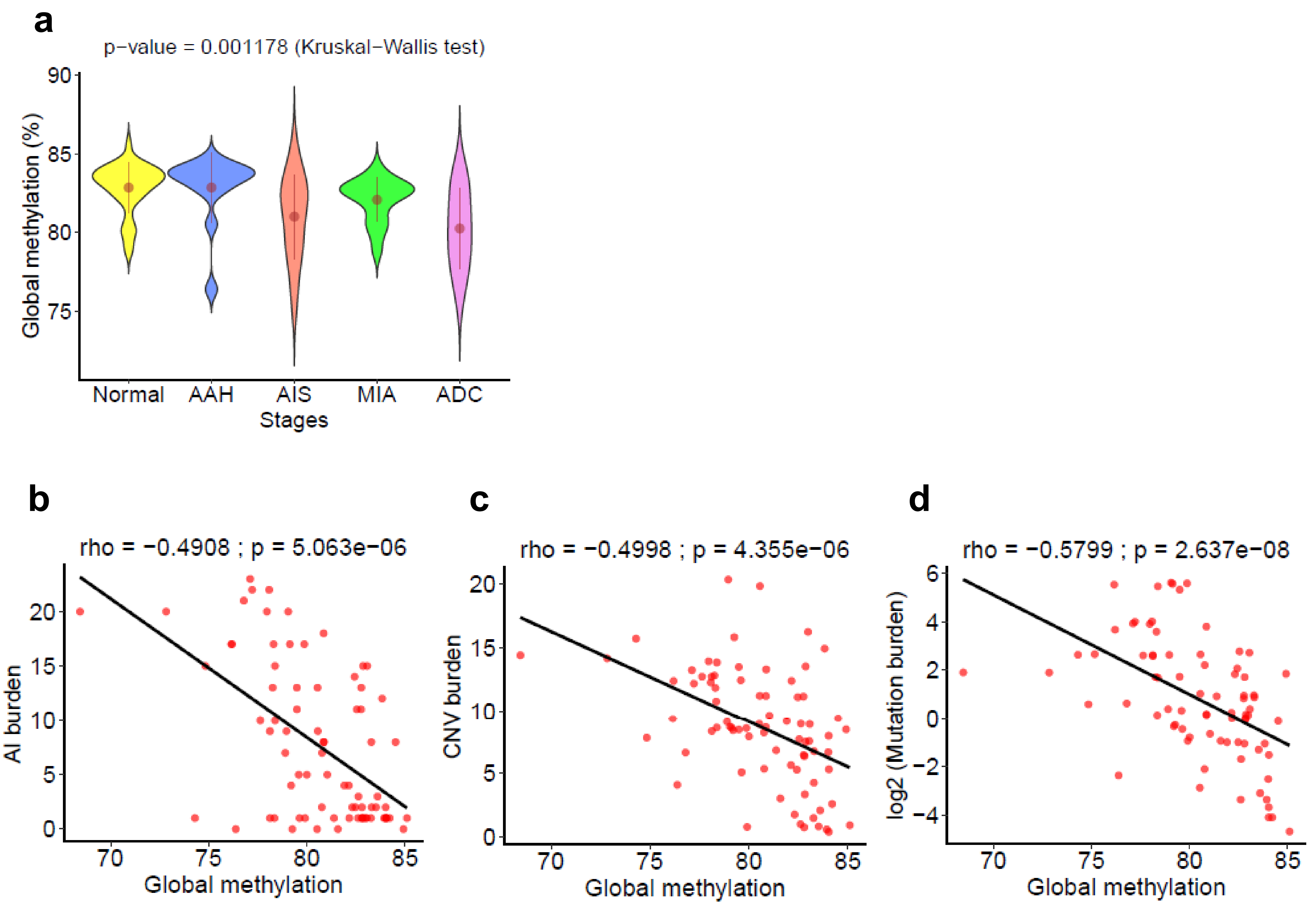

Supplementary Figure 12. Correlation of global methylation with genomic features in IPNs of different stages. (a) Global methylation level in IPNs of different stages using long interspersed transposable elements-1 (LINE-1) as a surrogate marker. The solid dots represent the means of global methylation level in IPNs of each histologic stage with error bars indicate 95% confidence intervals. The differences among all stages were assessed using two-sided Kruskal-Wallis H test. Correlation between global methylation levels and (b) number of events with allelic imbalance (AI), (c) percent of genes with copy number changes, (d) mutational burden (log2 transformed) assessed by two-tailed Spearman’s correlation analysis. Each dot represents each IPN specimen. Data from 35 patients was used in correlation analysis. (Source data is provided as a source data file.)

Supplementary Figure 13

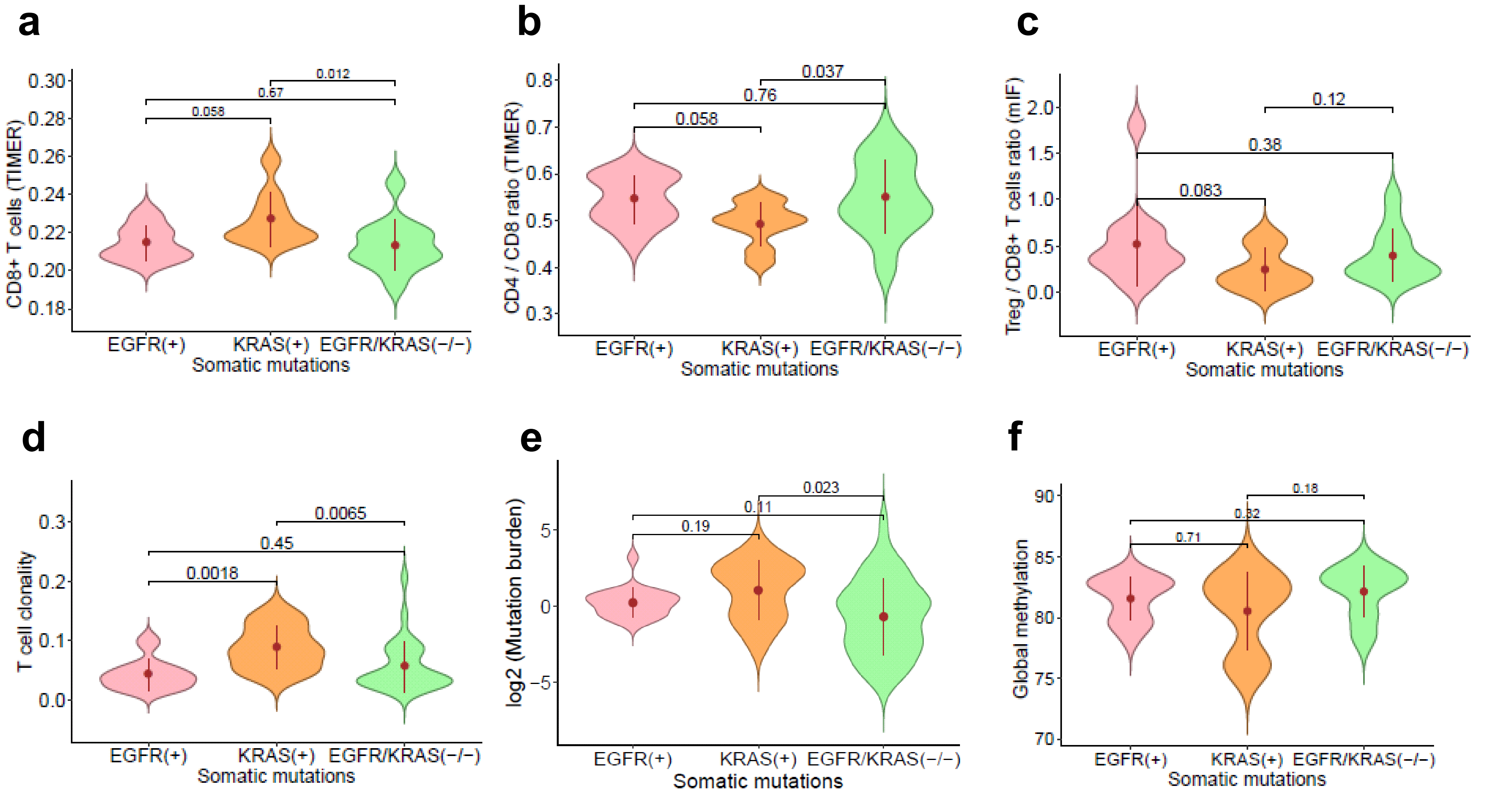

Supplementary Figure 13. The impact of oncogene mutations on T cell features and global genomic/methylation landscape. Comparison of infiltration of (a) CD8+ T cells, (b) CD4/CD8 ratio inferred from gene expression by TIMER, (c) Treg /CD8+CTL ratio by mIF, (d) T cell clonality by TCR sequencing, (e) mutation burden, (f) global methylation in IPNs with EGFR mutation (pink), KRAS mutation (orange) and wild-type for both KRAS and EGFR (green). The difference was assessed by two-sided Wilcoxon-rank sum test. Data from 35 patients was used in comparison. (Source data is provided as a source data file.)

Supplementary Table 1

Cell types	Markers
Total T cells	CD3+
CD4+ T cells	CD3+CD8-
Regulatory T cells	CD3+CD8-FoxP3+
CD8+ T cells	CD3+CD8+
CD8+ CTLs	CD3+CD8+Granzyme B+
ThCTLs	CD3+CD8-Granzyme B+

Supplementary Table 1. T cell subtypes defined by co-localized markers using multiplex Immunohistochemistry (mIF)
