# Supplementary material for: Immune evolution from preneoplasia to invasive lung adenocarcinomas and underlying molecular features
Source: Nat Commun. 2021 May 11;12:2722. doi: 10.1038/s41467-021-22890-x (PMC8113327; doi:10.1038/s41467-021-22890-x)
Supplement: Supplementary file 2 — Description of Additional Supplementary Files [file 41467_2021_22890_MOESM2_ESM.pdf]

Description of Additional Supplementary Files

File Name: Supplementary Data 1.

Description: Clinical characteristics and availability of different immune and molecular data

File Name: Supplementary Data 2.

Description: Gene Expression Profiling of NanoString® nCounter

File Name: Supplementary Data 3.

Description: Statistics of Differentially Expressed Genes
