# Supplementary material for: Immune evolution from preneoplasia to invasive lung adenocarcinomas and underlying molecular features
Source: Nat Commun. 2021 May 11;12:2722. doi: 10.1038/s41467-021-22890-x (PMC8113327; doi:10.1038/s41467-021-22890-x)
Supplement: Supplementary file 4 — Reporting Summary [file 41467_2021_22890_MOESM4_ESM.pdf]

nature research

Corresponding author(s): Jianjun Zhang
Last updated by author(s): Mar 1, 2021

Reporting Summary

Nature Research wishes to improve the reproducibility of the work that we publish. This form provides structure for consistency and transparency in reporting. For further information on Nature Research policies, see our Editorial Policies and the Editorial Policy Checklist.

Statistics

For all statistical analyses, confirm that the following items are present in the figure legend, table legend, main text, or Methods section.

n/a	Confirmed	
☐	☒	The exact sample size (*n*) for each experimental group/condition, given as a discrete number and unit of measurement
☐	☒	A statement on whether measurements were taken from distinct samples or whether the same sample was measured repeatedly
☐	☒	The statistical test(s) used AND whether they are one- or two-sided<br>*Only common tests should be described solely by name; describe more complex techniques in the Methods section.*
☐	☒	A description of all covariates tested
☐	☒	A description of any assumptions or corrections, such as tests of normality and adjustment for multiple comparisons
☐	☒	A full description of the statistical parameters including central tendency (e.g. means) or other basic estimates (e.g. regression coefficient) AND variation (e.g. standard deviation) or associated estimates of uncertainty (e.g. confidence intervals)
☐	☒	For null hypothesis testing, the test statistic (e.g. *F*, *t*, *r*) with confidence intervals, effect sizes, degrees of freedom and *P* value noted<br>*Give P values as exact values whenever suitable.*
☒	☐	For Bayesian analysis, information on the choice of priors and Markov chain Monte Carlo settings
☒	☐	For hierarchical and complex designs, identification of the appropriate level for tests and full reporting of outcomes
☐	☒	Estimates of effect sizes (e.g. Cohen's *d*, Pearson's *r*), indicating how they were calculated

Our web collection on statistics for biologists contains articles on many of the points above.

Software and code

Policy information about availability of computer code

Data collection	no software was used for data collection, we only performed analysis on resected pulmonary nodules (n = 64) and paired normal lung tissues (n = 52) from 52 patients, who underwent surgical resection.
Data analysis	nSolver 4.0 software was applied for nCounter® PanCancer Immune Profiling analysis; Ingenuity Pathway Analysis (IPA) software was applied for pathway analysis; TIMER1.0 was applied to infer the infiltration of immune cell subtypes. Vectra 3.0 multispectral microscope system, Phenochart1.0.9 viewer and Form 2.4.4 software were applied for Multiplex immunofluorescence staining and multispectral analysis. ImmunoSEQ Analyzer was applied for TCR sequencing analysis. ASCAT v2.5.2 was used to estimate tumor purity and ploidy. POLYSOLVER v1.0 was used to infer HLA alleles; LOHHLA was used to infer HLA loss. TrimGalore v.0.4.3 was used to trim Illumina adapter sequences; Bismark v.0.18.1 and bowtie2 v.2.2.3 was used to align the trimmed reads; FastQC v.0.11.7 was used for quality control. "stat_summary" and "geom_violin" function is from ggplot2 (v.0.9.1).

For manuscripts utilizing custom algorithms or software that are central to the research but not yet described in published literature, software must be made available to editors and reviewers. We strongly encourage code deposition in a community repository (e.g. GitHub). See the Nature Research guidelines for submitting code & software for further information.

Data

Policy information about availability of data

All manuscripts must include a data availability statement. This statement should provide the following information, where applicable:
- Accession codes, unique identifiers, or web links for publicly available datasets
- A list of figures that have associated raw data
- A description of any restrictions on data availability

The data for WES has been deposited at European Genome-phenome Archive (EGA), under the accession code: EGAS00001004960 [https://www.ebi.ac.uk/ega/

datasets/EGAD00001004960] and the data for RRBS is under EGAS00001004610, [https://www.ebi.ac.uk/ega/studies/EGAS00001004610]. All other data may be found within the main manuscript and supplementary information or available from the authors upon request. GSE102511 (Transcriptome sequencing of 15 normal lung parenchyma (NL), 17 atypical adenomatous hyperplasia (AAH) and 16 lung adenocarcinoma (LUAD) samples from 17 patients) was downloaded from GEO [https://www.ncbi.nlm.nih.gov/geo/query/acc.cgi?acc=GSE102511].

Field-specific reporting

Please select the one below that is the best fit for your research. If you are not sure, read the appropriate sections before making your selection.

- [x] Life sciences
- [ ] Behavioural & social sciences
- [ ] Ecological, evolutionary & environmental sciences

For a reference copy of the document with all sections, see nature.com/documents/nr-reporting-summary-flat.pdf

Life sciences study design

All studies must disclose on these points even when the disclosure is negative.

Sample size	Sample size was not determined before the study, rather based on sample availability. Because surgical resection is not the standard of care, there has been scarcity of resected specimens from lung adenocarcinoma precursors. The specimens were collected through international collaborations. All available samples were subjected to the analysis. In addition, the methylation aberrations in development and progression of lung precancers were unknown, therefore it was impossible to determine the power prior to RRBS profiling.
Data exclusions	No data were excluded from the analyses, as all samples was already subjected to rigorous QC by the sequence core. Since only a subset of samples are available for each analysis, we did analysis based on sample availability. 105 samples were used in TCRseq, 85 samples were used for gene expression profiling, 115 samples were used for WES, 70 samples were used for methylation profiling.
Replication	Due to scarcity of resected specimens from lung adenocarcinoma precursors, all available samples were subjected to the analysis. Replication was not feasible.
Randomization	This is not a study involving clinical trial, randomization does not apply to this study. Due to scarcity of resected specimens from lung adenocarcinoma precursors, all available samples were randomly subjected to nanoString gene expression profiling, TCRseq, WES, RRBS profiling and the corresponding analysis.
Blinding	This is not a study involving clinical trial, blinding does not apply to this study. And the analyst doesn't know the clinical information of the patients.

Reporting for specific materials, systems and methods

We require information from authors about some types of materials, experimental systems and methods used in many studies. Here, indicate whether each material, system or method listed is relevant to your study. If you are not sure if a list item applies to your research, read the appropriate section before selecting a response.

Materials & experimental systems

n/a	Involved in the study	
[ ]	[x]	Antibodies
[x]	[ ]	Eukaryotic cell lines
[x]	[ ]	Palaeontology and archaeology
[x]	[ ]	Animals and other organisms
[ ]	[x]	Human research participants
[x]	[ ]	Clinical data
[x]	[ ]	Dual use research of concern

Methods

n/a	Involved in the study	
[x]	[ ]	ChIP-seq
[x]	[ ]	Flow cytometry
[x]	[ ]	MRI-based neuroimaging

Antibodies

Antibodies used	pan-cytokeratin (AE1/AE3; epithelial marker; dilution 1:300; Dako, Carpinteria, CA, Cat# M3515), PD-L1 (clone E1L3N, dilution 1:100; Cell Signaling Technology, Beverly, MA, Cat# 13684), PD1 (clone EPR4877-2, dilution 1:250; Abcam, Cambridge, MA, Cat# ab137132), CD3 (T lymphocyte marker; dilution 1:100; Dako, Cat# GA50361-2), CD8 (cytotoxic T cell marker; clone C8/144B, dilution 1:20; Thermo Fisher Scientific, Waltham, MA, Cat# MA5-14548), and CD68 (macrophage marker; clone PG-M1, dilution 1:450; Dako, Cat# GA60961-2); and Panel 2 contained pan-cytokeratin, CD3, CD8, CD45RO (memory T cell marker; clone UCHL1, Leica Biosystems, Buffalo Grove, IL, Cat# PA0146), Granzyme B (cytotoxic lymphocyte marker; clone F1, ready to use; Leica Biosystems, Cat# PA0291), and FoxP3 (regulatory T cell marker; clone 206D, dilution 1:50; BioLegend, SanDiego, CA, Cat# 126404).
Validation	pancytokeratin: https://www.citeab.com/antibodies/2414780-m3515-cytokeratin-concentrate<br>PD-L1: https://www.cellsignal.com/science-resources/pd-li-signaling/pd-li<br>PD1: https://www.abcam.com/pd1-antibody-panel-epr48772-sp269-cal20-nat105-nat105-epr21106-ab252192.html<br>CD3: https://www.agilent.com/en/product/immunohistochemistry/antibodies-controls/primary-antibodies/cd3-(dako-omnis)-76197

CD8: https://www.thermofisher.com/antibody/product/CD8-Antibody-clone-SP16-Monoclonal/MA5-14548
CD68: https://www.agilent.com/en/product/immunohistochemistry/antibodies-controls/primary-antibodies/cd68-(dako-omnis)-76223
CD45RO: https://shop.leicabiosystems.com/us/ihc-ish/ihc-primary-antibodies/pid-cd45ro
Granzyme B: https://shop.leicabiosystems.com/us/ihc-ish/ihc-primary-antibodies/pid-granzyme-b
FOXP3: https://www.biolegend.com/en-us/products/pe-anti-mouse-foxp3-antibody-4660?GroupID=GROUP20

Human research participants

Policy information about studies involving human research participants

Population characteristics	All patients are from China and Japan, with median age of 69.8 (range 44-80.5) and the ratio of male to female is 22:17. All patients presented as pulmonary nodules and treated with upfront surgical resection. None of the patients received neoadjuvant treatment.
Recruitment	No patients were recruited specifically for this study. All patients were treated as standard of care and surgical specimens were randomly collected and analyzed. No self-selection process is involved.
Ethics oversight	Written informed consent was obtained from all patients involved. The study was approved by institutional review board (IRB) from Nagasaki University Hospital, Zhejiang Cancer Hospital and MD Anderson Cancer Center. This study is compliant with the "Guidance of the Ministry of Science and Technology (MOST) for the Review and Approval of Human Genetic Resources", which requires formal approval for the export of human genetic material or data from China.

Note that full information on the approval of the study protocol must also be provided in the manuscript.
